# A DFT study on the degradation mechanism of vitamin B2

**DOI:** 10.1016/j.fochms.2022.100080

**Published:** 2022-01-28

**Authors:** Shinichi Yamabe, Noriko Tsuchida, Shoko Yamazaki

**Affiliations:** a Department of Chemistry, Nara University of Education, Takabatake-cho, Nara 630-8528, Japan; b Department of Liberal Arts, Faculty of Medicine, Saitama Medical University, 38 Morohongo, Moroyama-machi, Iruma-gun, Saitama 350-0495, Japan

**Keywords:** Vitamin B2, Riboflavin, Degradation mechanism, Density functional theory calculations, Transition states

## Abstract

Degradation reaction paths starting from riboflavin (RF) were investigated using DFT (density functional theory) as the first attempt to reveal their elementary processes. Photochemical reactions were followed in the lowest triplet spin state, “(T)”. Two intermediates [Int1(T) and Int2(T)] were found in the course, RF(T) → FMF (7,8-dimethyl-10-formylflavin, T). From FMF(T), there are two degradation channels. Release of ketene(T) and carbon monoxide leads to LC (lumichrome, S_0_) and LF (lumiflavin, T), respectively. The base-catalyzed (ground state) degradation of FMF was investigated with HO^–^(H_2_O)_3_. The Grotthuss-type proton transfer along hydrogen bonds controlled the degradation reaction. All the transition states of cleavage of C—C and C—N covalent bonds were determined, and the degradation mechanism was clarified.

## Highlights

• Elementary processes of degradation from riboflavin were found by DFT calculations.
• Photochemical reaction courses in the lowest triplet spin state were elucidated.
• Base-catalyzed degradation paths from formylmethylflavin were determined.
• All the transition states of cleavage of C-C and C-N covalent bonds were determined.

## Graphical abstract

**Figure:**
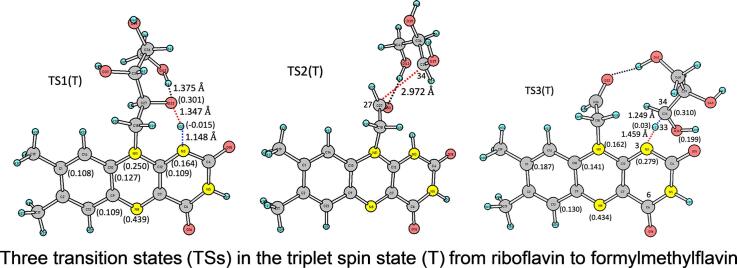

## 1 Introduction

**Scheme 1:**
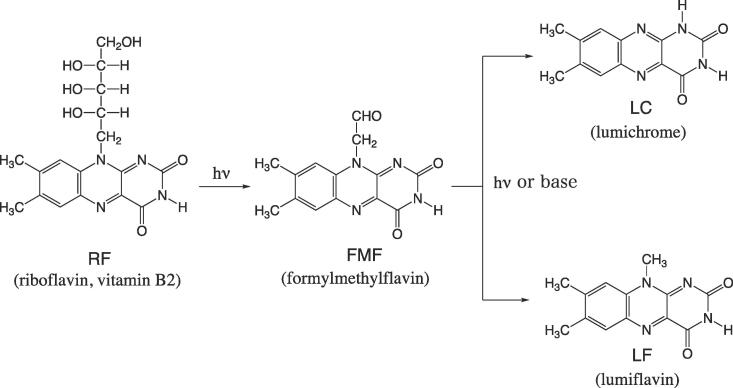
The photo-degradation of RF to LC and LF via FMF and the base catalyzed reaction of FMF to LC and LF.

Riboflavin (RF) or vitamin B2 is a water-soluble vitamin that is widely present in a variety of foods, such as almonds, mushrooms and vegetables (Ball, 2006). Photo, thermal, and chemical degradation reactions of the vitamin are serious problems for its preservation (Uchida, et al., 2016). A general scheme for the degradation of RF is shown in Scheme 1 (Smith and Metzler, 1963, Ali et al., 2014, Song et al., 1965).

Aqueous solutions of riboflavin (RF) are unstable in light (hν) and degrade to give formylmethylflavin (FMF), lumiflavin (LF) and lumichrome (LC) (Ahmad et al., 2004, Huang et al., 2006). While FMF is the intermediate in the photo-degradation, it can also lead to LF and LC in the dark (non-photochemical) basic aqueous solution (Song et al., 1965, Ahmad et al., 1980). The kinetics of both photolysis of RF (Woodcock et al., 1982, Allen and Parks, 1979: Ahmad et al., 2019, Gul et al., 2014, Ahmad et al., 2004, Ahmad et al., 2010, Ahmad et al., 2013, Ahmad et al., 2008) and basic hydrolysis of FMF (Song et al., 1965, Ahmad et al., 1980) have been studied extensively including gas-phase photofragmentation studies of deprotonated riboflavin, [RF − H]^−^, using laser-interfaced mass spectrometry (Wong, et al., 2021). O—H bond dissociation energies of the phenolic compounds (rutin and catechin) and T_1_ state H-atom affinity of RF were evaluated to examine the role of polyphenolic antioxidants against the photo-oxidative damage induced by RF (Ji and Shen, 2008). However, mechanisms of C—C and C—N covalent bond cleavage in RF and FMF are still unclear. Precise understanding of the mode of the bond scission in the ribityl side chain during photo- and thermal degradations would contribute to development of methods for stabilizing the vitamin B2 in products exposed to both light and heat during preparation and storage, or retail.

Since there have been no theoretical studies of RF degradation, in this work, DFT (density functional theory) calculations of reaction paths in Scheme 1 were carried out. Elementary processes were traced and the intervening species, which have not been detected experimentally, were investigated. The following questions were scrutinized carefully: (1) photo-irradiation of RF leads to the (π, π*) excited state of the tricyclic isoalloxazine ring (Sikorska, et al., 2005), because the HOMO (highest occupied molecular orbital) and LUMO (lowest unoccupied orbital) are π and π* orbitals and the state appears not to be concerned with cleavage of σ bonds in the ribityl side chain [–CH(OH)–CH(OH)–CH(OH)–CH_2_OH], thus it is not clear how (π → π*) excitation is related to the scission of C–C σ bond; and (2) whether photochemical and base-catalyzed reactions of FMF → LF + LC (in Scheme 1) are similar or not.

## 2 Methods of calculations

Riboflavin is a water soluble species, thus hydrogen bonds with water molecules that induce proton transfer need to be included explicitly in the reaction model. Thanthiriwatte et al. (2011) conducted extensive investigations of various hydrogen-bonded dimers, using DFT potential energy curves. Comparisons between these and CBS extrapolated limit CCSD(T) ones were made, where CBS stands for complete basis set and CCSD(T) stands for coupled cluster calculations, using both single and double substitutions from the Hartree-Fock determinant with triple excitations. The functional wB97X-D (Chai and Head-Gordon, 2008) which includes the dispersion correction provides very good potential energy curves, therefore it was used in this work. Geometry optimizations of reaction systems were carried out including the PCM (polarizable continuum model, Cances et al., 1997, Cossi et al., 1998, Mennucci and Tomasi, 1997) solvent effect. The adopted basis set was 6–311 + G(d,p). Reaction paths in the excited state were traced by the use of the unrestricted wB97X-D, i.e., UwB97X-D, in the triplet spin state. This is because reactions in Scheme 1 are known to occur in the spin state after the intersystem crossing from the singlet excited state (Ali, et al., 2014). In addition, the spin unrestricted DFT was reported to give the energy gap between the ground state and the lowest triplet state of RF, which was in good agreement with that derived from the emission spectra (Sikorska, et al., 2005).

First, transition states (TSs) were sought by partial optimizations at bond interchange regions. Second, by the use of Hessian matrices, TS geometries were optimized. They were characterized by vibrational analyses, which checked whether the obtained geometries had single imaginary frequencies (ν^‡^s). From TSs, reaction paths were traced by the intrinsic reaction coordinate (IRC) method (Fukui, 1981, Hratchian and Schlegel, 2005) to obtain the energy-minimum geometries.

Energy changes were shown by the use of Gibbs free energies (T = 298.15 K, P = 1 atm). All the calculations were carried out using the GAUSSIAN 16 (Frisch et al., 2016) program package. The computations were performed at the Research Center for Computational Science, Okazaki, Japan. All the Cartesian coordinates of the calculated species are shown in the Supplementary data.

## 3 Results and discussion

### 3.1 The fragmentation of RF to FMF in the triplet spin state

**Scheme 2:**
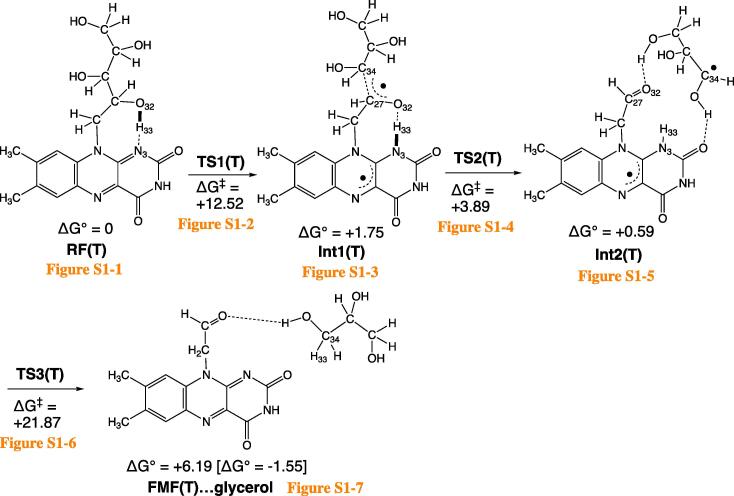
Calculated channels of the degradation of RF. “(T)” stands for the triplet spin state. Optimized geometries are shown in Fig. S1. Changes of Gibbs free energies, ΔG, are in kcal/mol. To the product, FMF(T)…glycerol, [ΔG° = −1.55 kcal/mol] is assigned, which was obtained by ΔG° = G°(FMF(T)) + G°(glycerol) – G°(RF(T)).

Scheme 2 shows the fragmentation path of RF(T) → FMF(T), where (T) stands for the triplet spin state. Each optimized geometry is shown in Fig. S1.

**Scheme 3:**
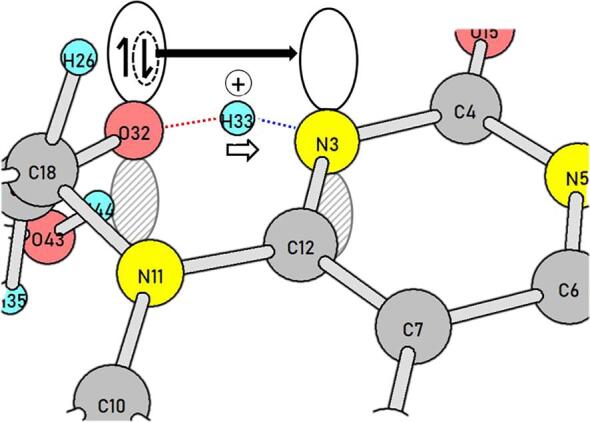
Concomitant migration of an electron (the bold rightward arrow) and the proton H(33) conveys the radical character to the ribityl side chain.

Among various conformations of the ribityl side chain in RF, that with the O(32)-H(33)…N(3) intramolecular hydrogen bond was adopted as the precursor geometry (Fig. S1-1). Along the hydrogen bond, the H(33) migration TS, TS1(T), is obtained (Fig. S1-2). Noteworthy is that H(33) is not a hydrogen atom but a proton, and concomitantly one electron is shifted along O(32) → N(3). The shifts of the proton and one electron are illustrated in Scheme 3.

After TS1(T), the first intermediate, Int1(T) (Fig. S1-3), is afforded. Its C(27)-C(34) bond is elongated (1.681 Å) and is ready for scission. At TS2(T) (Fig. S1-4), the bond is cleaving, and a radical-pair Int2(T) (Fig. S1-5) is yielded. From Int2(T), the N(3)-H(33) bond is converted to the H(33)-C(34) bond at TS3(T) (Fig. S1-6). The conversion follows the pattern of Scheme 3, i.e., the concomitant proton H(33) and one electron shift. To convey one electron from the tricyclic ring, the shifts occur out of the plane. In fact, the angle ∠H(33)-N(3)-C(6) is 110.71° at TS3(T). The out-of-plane bending of the N(3)-H(33) bond is attained by the 34th harmonic vibration with ν = 689.07 cm^−1^ of which the mode vector is completely localized at the N(3)-H(33) bond (Fig. S2). After TS3(T), a complex of FMF(T) and glycerol HO-CH_2_-CH(OH)–CH_2_OH (Fig. S1-7) is generated. In Int2(T), two radical centers are separated, and the open singlet state Int2(S) is also likely. The conversion of [Int2(S) → FMF(S) + glycerol] takes place without an energy barrier.

The transient radical-pair intermediate, Int2(T), is suggested here for the (RF → FMF) degradation. Int2(T) is composed of FMF-H• and HOCH_2_-C(OH)H-C(OH)H•. These two radicals are bound by two C=O…HO hydrogen bonds. The dual hydrogen-bond restriction blocks separation of these radicals.

### 3.2 The reaction of FMF → LC and LF in the triplet state

**Scheme 4:**
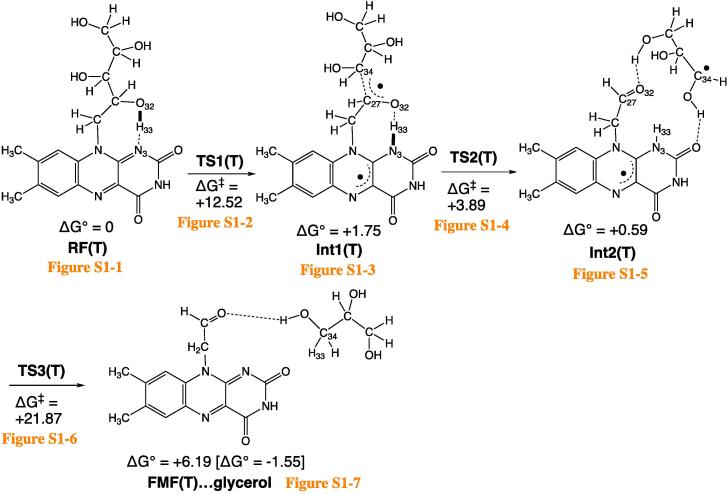
Calculated paths of the degradation of FMF(T) to LC(T) and LF(T) (in the triplet spin state). The optimized geometries are shown in Fig. S3 (to LC) and in Fig. S4 (to LF), respectively. ΔG values are relative to the energy of RF(T) in Fig. S1-1. For instance, ΔG° = −1.55 kcal/mol of FMF(T) was obtained by G°(FMF(T)) + G°(glycerol) – G°(RF(T)).

Scheme 4 exhibits degradation paths of FMF(T) → LC(T) and LF(T).

TS4(T) (Fig. S3-2) shows the H(32) migration TS for conversion of C(26)-H(32) to H(32)-N(3). The migration pattern is similar to that of TS1(T) in Fig. S1-2. H(32) is moving as a proton and concomitantly one electron is moving from C(26) to N(3) in the π electronic cloud. After TS4(T), Int3(T) (Fig. S3-3) is formed. From Int3(T), cleavage of the N(11)-C(18) bond is brought about at TS5(T) (Fig. S3-4). After TS5(T), triplet ketene (H_2_C = C = O(T)) is evolved and the ground state LC(S_0_) is afforded as shown in Fig. S3-5. In spite of the stability of the triplet state in the tricyclic ring of LC, the ketene becomes the triplet state. In fact, the [LC(S_0_) + ketene(T)] system is 8.03 kcal/mol more stable than the [LC(T) + ketene(S_0_)] one.

TS6(T) (Fig. S4-1) is the C…C separation TS, which gives Int4(T) (Fig. S4-2) containing the formyl radical H-C•=O. From the radical pair Int4(T), the H(26) migration takes place at TS7(T) (Fig. S4-3), leading to LF(T) and CO (Fig. S4-4). The triplet state of Int4(T) has two distant unpaired electrons (at the tricyclic ring and at the formyl radical), and the open singlet spin state would be formed readily. In this state, the H(26) migration forming the methyl group occurs without an energy barrier.

The FMF(T) → LC + H_2_C=C=O(T) route has small activation free energies, ΔG^‡^ = +16.61 kcal/mol of TS4(T) and ΔG^‡^ = +18.03 kcal/mol of TS5(T), while the reaction free energy [ΔG° = −4.75 kcal/mol] is of poor exothermicity. On the other hand, the FMF(T) → LF(T) + CO route has large activation free energies, ΔG^‡^ = +34.98 kcal/mol of TS6(T) and ΔG^‡^ = +39.79 kcal/mol of TS7(T), while the reaction free energy [-13.32 kcal/mol] is of large exothermicity. Thus, the FMF(T) → LC + H_2_C=C=O(T) route is kinetically favorable, and the FMF(T) → LF(T) + CO route is thermodynamically favorable.

In the reference (Martin, et al., 2002), reaction energies of the hydrogen-atom transfer of LF(T) with a model tetrahydrofuran, (*R*)-2-amino-(*S*)-4-hydroxy-(*R*)-5-(hydroxymethyl)-tetrahydrofuran, “subst.THF” here, were calculated by the gas-phase B3LYP/6–31 + G**. Abstraction of the α hydrogen of subst.THF was shown to be of the largest exothermicity (in Fig. 2 of the reference). Since the transition state of the abstraction was not reported in the reference, it was determined in this study by uwB97xd/6–311 + G(d,p) SCRF=(PCM, solvent = water). The calculated result is shown in Fig. S6. The ΔG^‡^ value is small, +10.11 kcal/mol. Accordingly, LF(T) was confirmed to be of a high reactivity for the hydrogen abstraction.

### 3.3 The base-catalyzed reaction of FMF → LC and LF

**Scheme 5:**
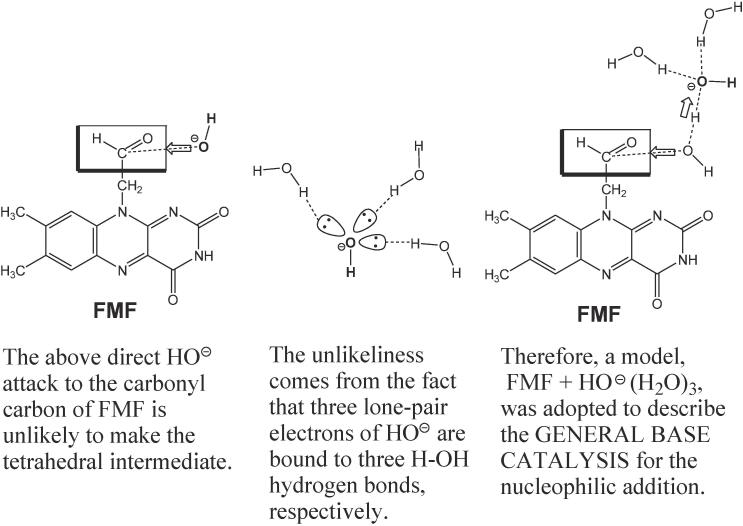
An explanation for employment of the reaction model, FMF + HO^–^(H_2_O)_3_.

To simulate the reaction, a model of FMF + HO^–^(H_2_O)_3_ was adopted as illustrated in Scheme 5. In the scheme, the first step consisting of the HO addition to the terminal aldehyde group of FMF is expressed by the general base catalysis (Jencks and Carriuolo, 1961).

**Scheme 6:**
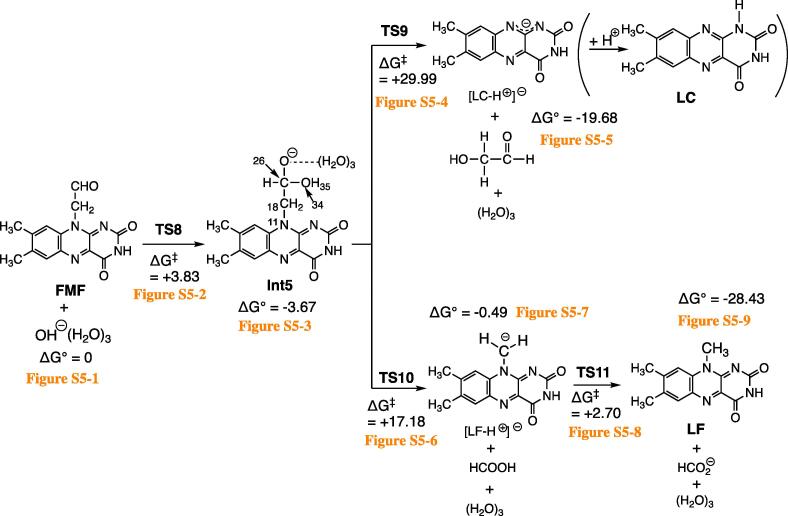
The calculated paths of the base-catalyzed degradation of FMF to LC and LF. The optimized geometries are shown in Fig. S5.

Scheme 6 shows the calculated results.

**Scheme 7:**
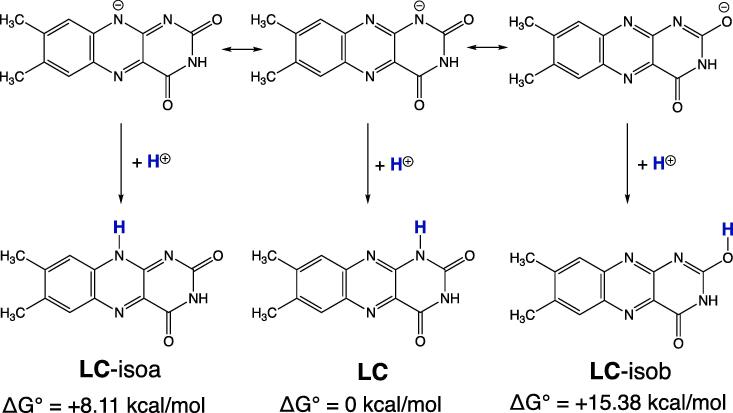
Three canonical resonance structures of [LC-H^+^]^−^ and the corresponding protonated (neutral) isomers. Those with positive values of ΔG° are less stable. LC-isoa and LC-isob stand for isomers of LC.

The tetrahedral intermediate Int5 (Fig. S5-3) is formed via the general base catalysis, i.e., TS8 (Fig. S5-2). From Int5, two TSs are found. One is TS9 (Fig. S5-4), where O(34)-H(35) is shifted from C(26) to C(18) and simultaneously the C(18)---N(11) bond is cleaved. After TS9, the deprotonated LC, [LC-H^+^]^−^, HO-CH_2_-CH=O (glycolaldehyde) and (H_2_O)_3_ are produced (Fig. S5-5). The subsequent step to LC + HO-CH_2_-CH=O + HO^–^(H_2_O)_2_ could not be obtained. Therefore, [LC-H^+^]^−^ is the product under basic (pH > 7) conditions. LC would be obtained by addition of the acid as described as “The hydrolyzed solutions of FMF were buffered to pH 2” (Ahmad, et al., 1980). The anion [LC-H^+^]^−^ has three canonical resonance structures as shown at the upper part of Scheme 7.

Therefore, three isomers are possible after the protonation to the anion. LC is confirmed to be the most stable relative to LC-isoa and LC-isob.

The other TS from Int5 is TS10 (Fig. S5-6), which gives [LF-H^+^]^−^ + H_2_CO_2_ (formic acid) + (H_2_O)_3_ in Fig. S5-7. The exocyclic carbanion of [LF-H^+^]^−^ is subject to protonation at TS11 (Fig. S5-8). It is a Grotthuss-type proton transfer (de Grotthuss, 1806) starting from formic acid. After TS11, LF, HCO_2_^–^ (formate ion) and (H_2_O)_3_ are afforded.

ΔG^‡^ = +17.18 kcal/mol of TS10 leading to LF is much smaller than ΔG^‡^ = +29.99 kcal/mol of TS9 leading to LC. In fact, the base-catalyzed reaction of FMF gave mainly LF with a less significant competing reaction yielding LC (Song, et al., 1965).

The reason why two fragmentation channels are present from Int5 is explained as follows. The tetrahedral intermediate Int5 has the unstable alkoxide moiety -(H)(OH)C-O^−^ and its recovery to the carbonyl group C=O is required. There are two pathways for the recovery. One is TS9 where the N(11)-C(18) cleavage and OH migration occur simultaneously to form HOCH_2_-C(=O)H. The other is TS10 where the C(18)-C(26) cleavage gives H-C(=O)OH.

## 4 Concluding remarks

In this work, degradation reaction paths starting from riboflavin (RF) were investigated by DFT calculations for the first time. In the route from RF to FMF of the lowest triplet spin state (Scheme 2), two intermediates Int1(T) and Int2(T) were found. Int1(T) was formed by the concomitant transfer of one proton and one electron of the hydroxyl group of the ribityl side chain. The terminal aldehyde group of FMF (formylmethylflavin) was afforded in Int2(T) after the C…C cleavage. From FMF(T), there were two degradation channels (Scheme 4). Release of ketene(T) and carbon monoxide led to LC (lumichrome, S_0_) and LF (lumiflavin, T), respectively.

Question (1) has been raised in the Introduction, i.e., the relation between the (π, π*) triplet state and the cleavage of σ bonds. As shown in TS1(T) (Fig. S1-2) and TS4(T) (Fig. S3-2), the in-plane migration of a proton and the out-of-plane one of an electron occurred simultaneously. The migration weakened a σ bond in the ribityl chain, which gave rise to the degradation.

The base-catalyzed (ground state) degradation of FMF was investigated with HO^–^(H_2_O)_3_ (Scheme 6). The first step was formation of the tetrahedral intermediate Int5 (Fig. S5-3). Scission of N-C and C-C bonds in the –CH_2_-CH(OH)-O^−^ group of Int5 led to [LC-H^+^]^−^ and [LF-H^+^]^−^, respectively.

Question (2) in the Introduction is about the similarity or the difference between the photochemical reaction of FMF to LC and LF and the ground base-catalyzed one. While the driving force was different, cleavage of N-C and C-C bonds occurred in a similar way.

## Declaration of Competing Interest

The authors declare that they have no known competing financial interests or personal relationships that could have appeared to influence the work reported in this paper.
