# Supplementary material for: A DFT study on the degradation mechanism of vitamin B2
Source: Food Chem (Oxf). 2022 Jan 28;4:100080. doi: 10.1016/j.fochms.2022.100080 (PMC8991990; doi:10.1016/j.fochms.2022.100080)
Supplement: Supplementary data 1 [file mmc1.pdf]

A DFT study on the degradation mechanism of vitamin B2

Shinichi Yamabe,[a] Noriko Tsuchida,[b] and Shoko Yamazaki*[a]

[a]*Department of Chemistry, Nara University of Education, Takabatake-cho, Nara 630-8528, Japan*

[b]*Department of Liberal Arts, Faculty of Medicine, Saitama Medical University, 38 Morohongo, Moroyama-machi, Iruma-gun, Saitama 350-0495, Japan*

email: yamazaks@cc.nara-edu.ac.jp

Figure S1. Geometries of species in Scheme 2 optimized by uwB97x-D/6-311+G(d,p) SCRF=(PCM,solvent=water). From RF(T) to FMF(T) and glycerol. Pages S2-4

Figure S2. The 34-th normal vibration of the [FMF+H•] radical which is the fragment of Int2(T) in Figure S1-5. Page S5

Figure S3. Geometries of species in Scheme 4. From FMF(T) to LC + ketene(T). Pages S6-7

Figure S4. Geometries of species in Scheme 4. From FMF(T) to LF(T) + carbon monoxide. Pages S8-9

Figure S5. Geometries of species in Scheme 6. Pages S10-13

Figure S6. A hydrogen-shift reaction between the triplet lumiflavin, LF(T), and (*R*)-2-amino-(*S*)-4-hydroxy-(*R*)-5-(hydroxymethyl)tetrahydrofuran. Pages S14

An input data, Cartesian coordinates and energies Pages S15-44

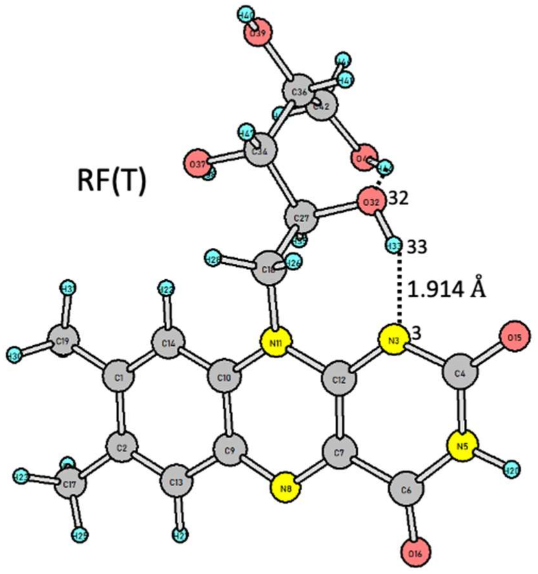

Fig. S1-1

ΔG° = 0 kcal/mol

↓

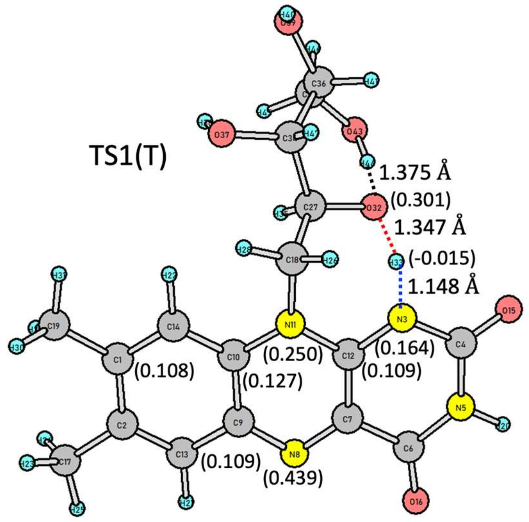

Fig. S1-2

$\Delta G^{\ddagger}$ = +12.52 kcal/mol

$\nu^{\ddagger}$ = 1376.9802i cm^{-1}

↓

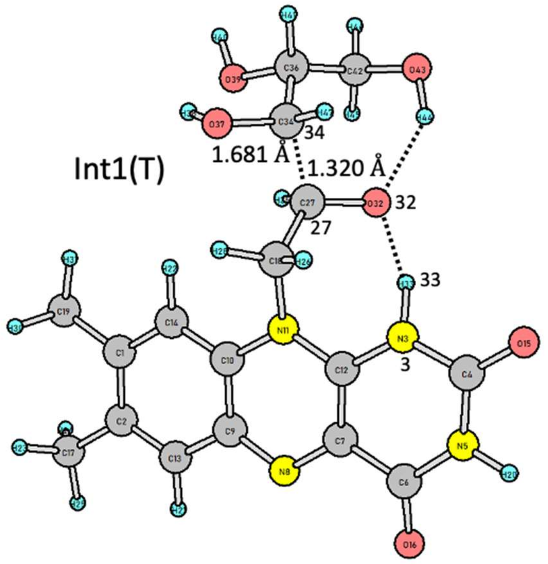

Fig. S1-3

ΔG° = +1.75 kcal/mol

↓

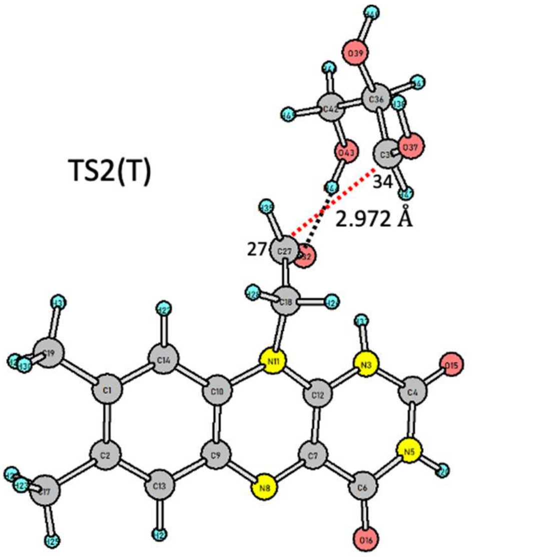

Fig. S1-4
ΔG‡ = +3.89 kcal/mol
ν‡ = 20.5900*i* cm^{-1}

↓

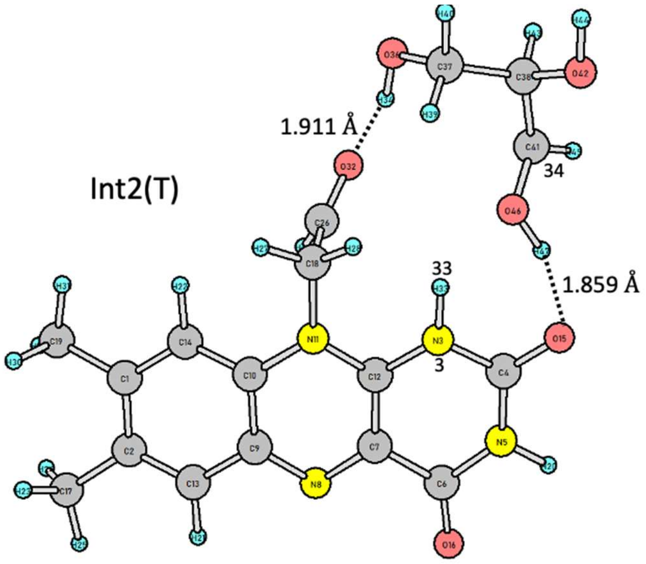

Fig. S1-5
ΔG° = +0.59 kcal/mol

↓

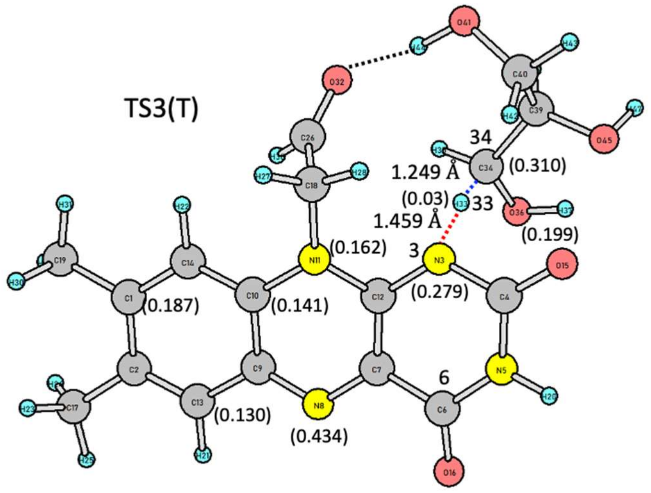

Fig. S1-6
ΔG‡ = +21.87 kcal/mol
ν‡ = 1212.2537*i* cm^{-1}

↓

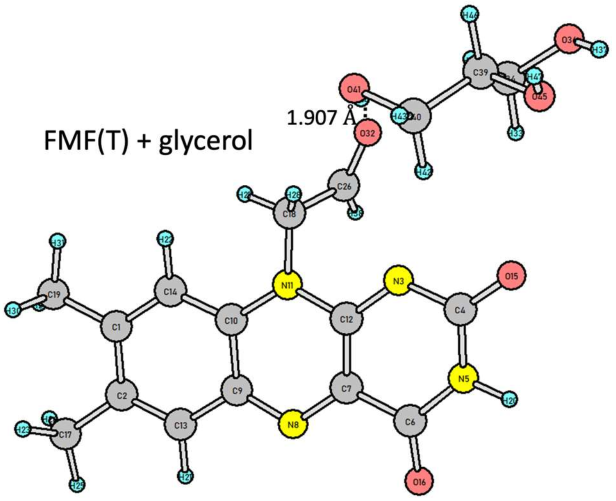

Fig. S1-7
ΔG° = +6.19 kcal/mol
[ΔG° = -1.55 kcal/mol]

Figure S1. Geometries of species in Scheme 2 optimized by uwB97x-D/6-311+G(d,p) SCRF=(PCM,solvent=water). From RF(T) to FMF(T) and glycerol. Their Cartesian coordinates and energies are in Supporting Information. At TSs, red and blue broken arrows stand for covalent bonds cleaved and formed, respectively. Values in parentheses are Mulliken spin densities.

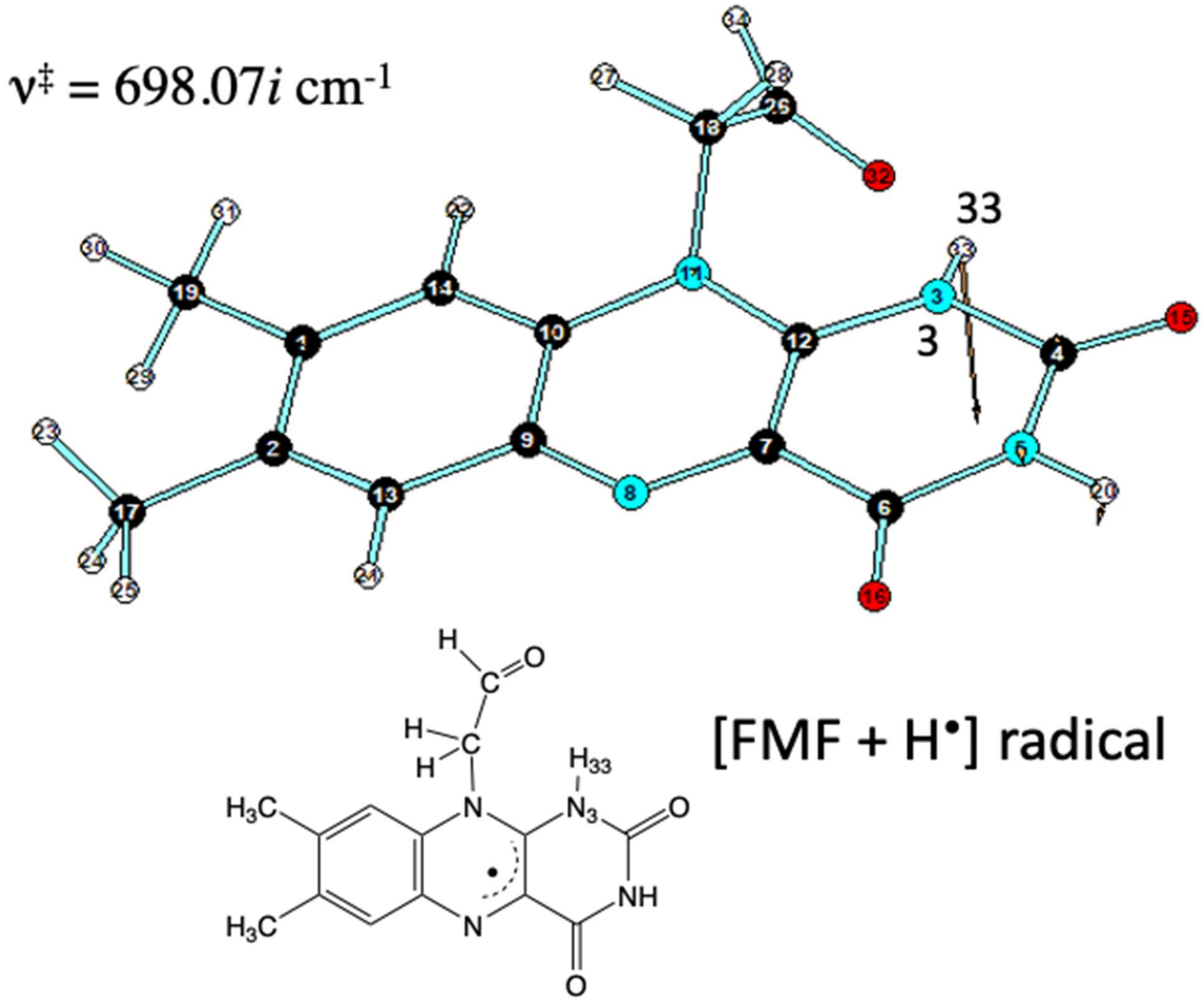

Figure S2. The 34-th normal vibration of the [FMF+H•] radical which is the fragment of Int2(T) in Figure S1-5.

FMF(T)

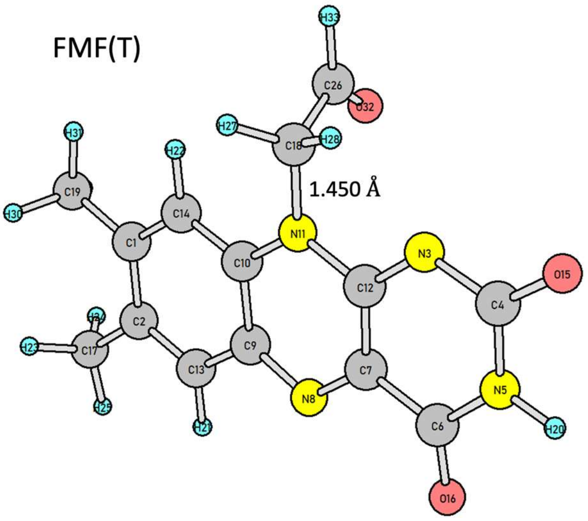

Fig. S3-1

ΔG° = -1.55 kcal/mol

↓

TS4(T)

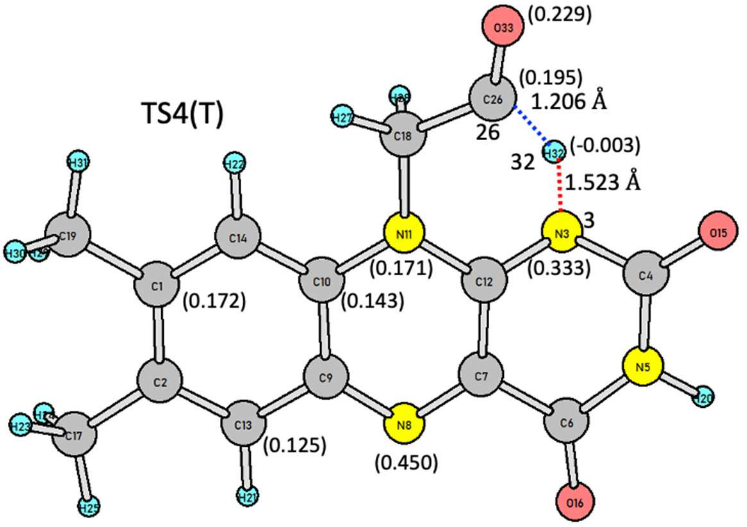

Fig. S3-2

$\Delta G^{\ddagger} = +16.61$ kcal/mol

$\nu^{\ddagger} = 1231.5711i$ cm^{-1}

↓

Int3(T)

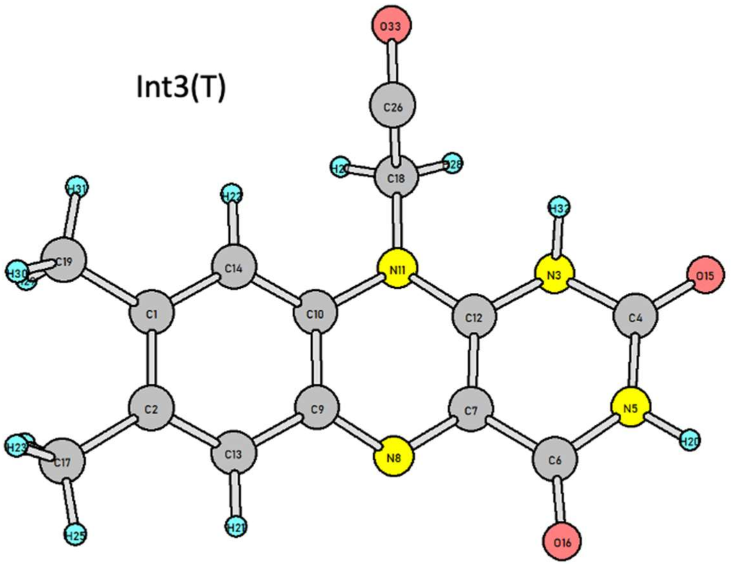

Fig. S3-3

ΔG° = -7.82 kcal/mol

↓

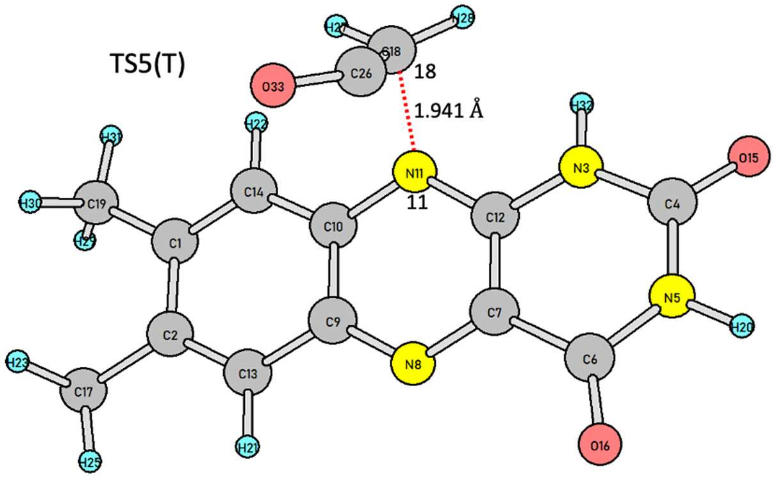

Fig. S3-4
$\Delta G^{\ddagger}$ = +18.03 kcal/mol
$\nu^{\ddagger}$ = 727.8287*i* cm^{-1}

↓

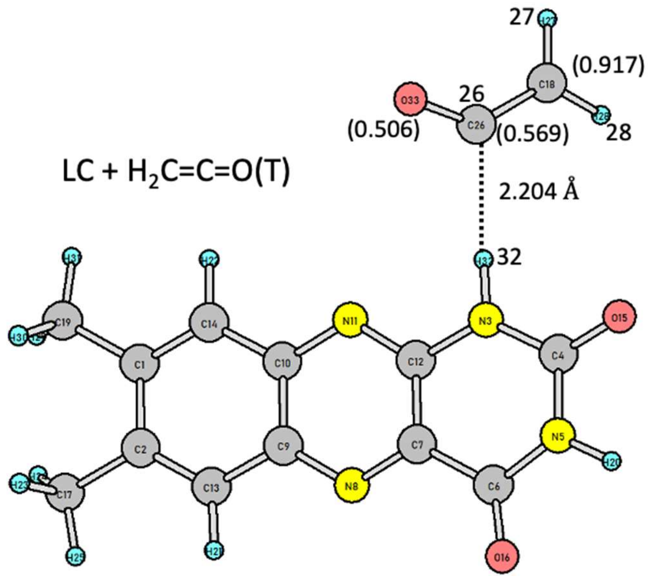

Fig. S3-5
ΔG° = +1.12 kcal/mol
[ΔG° = -4.75 kcal/mol]

Figure S3. Geometries of species in Scheme 4. From FMF(T) to LC + ketene(T). ΔG° values are relative to the energy of G°(RF(T))-G°(glycerol).

FMF(T) in Figure S3-1

↓

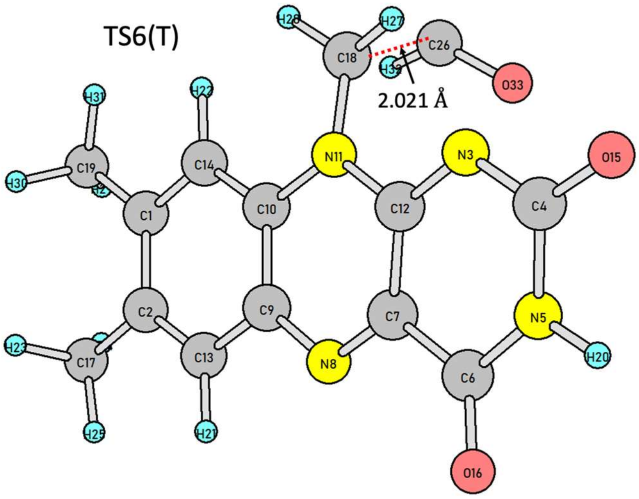

Fig. S4-1
$\Delta G^{\ddagger}$ = +34.98 kcal/mol
$\nu^{\ddagger}$ = 416.8362i cm^{-1}

↓

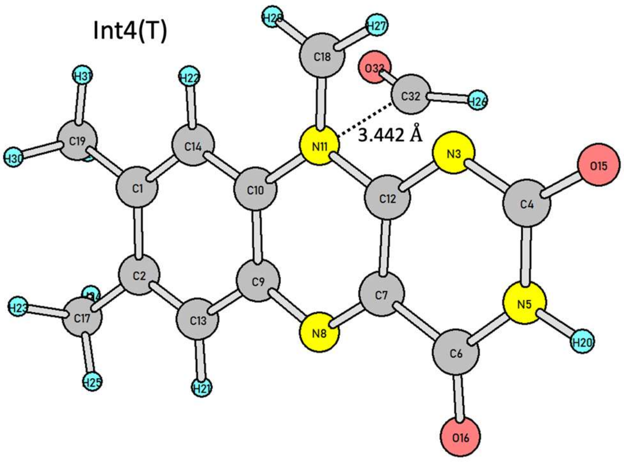

Fig. S4-2
ΔG° = +20.43 kcal/mol

↓

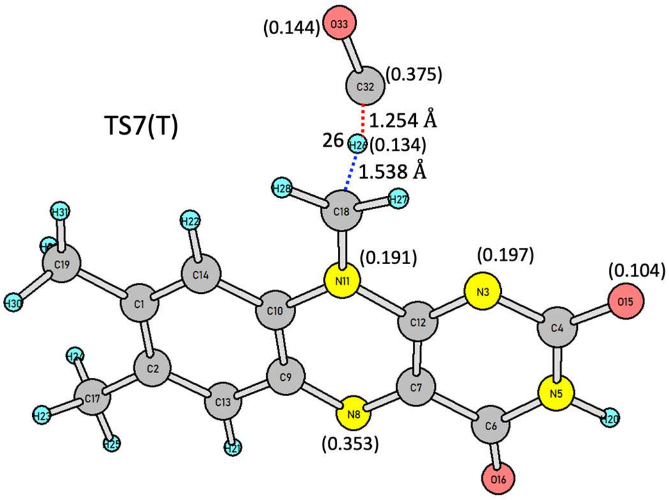

Fig. S4-3
$\Delta G^{\ddagger}$ = +39.79 kcal/mol
$\nu^{\ddagger}$ = 1950.9671i cm^{-1}

↓

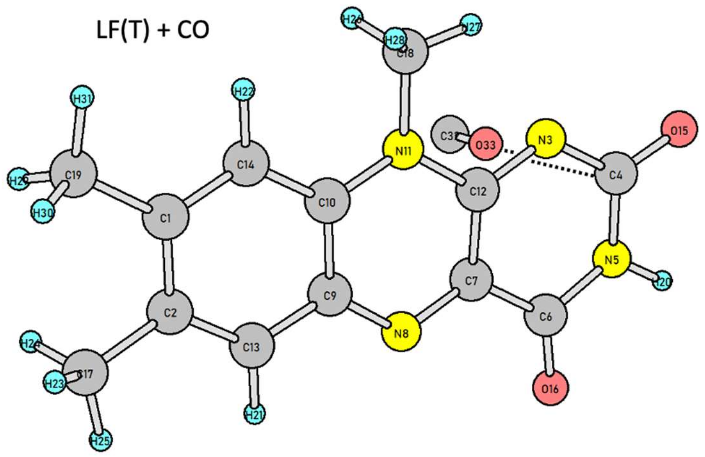

Fig. S4-4
ΔG° = -5.92 kcal/mol
[ΔG° = -13.32 kcal/mol]

Figure S4. Geometries of species in Scheme 4. From FMF(T) to LF(T) + carbon monoxide.

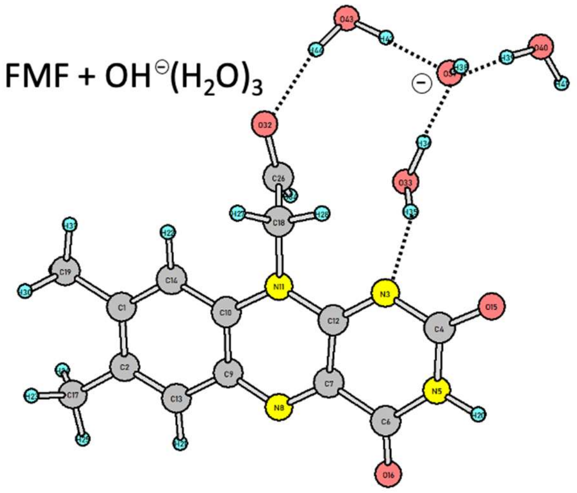

Fig. S5-1
$\Delta G^{\circ} = 0$ kcal/mol

↓

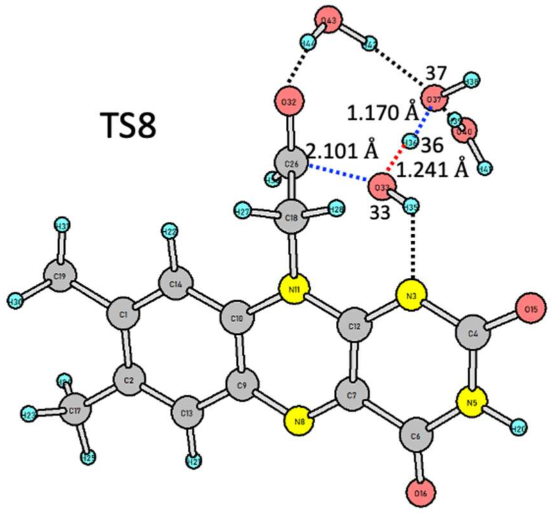

Fig. S5-2
$\Delta G^{\ddagger} = +3.83$ kcal/mol
$\nu^{\ddagger} = 751.2610i$ cm^{-1}

↓

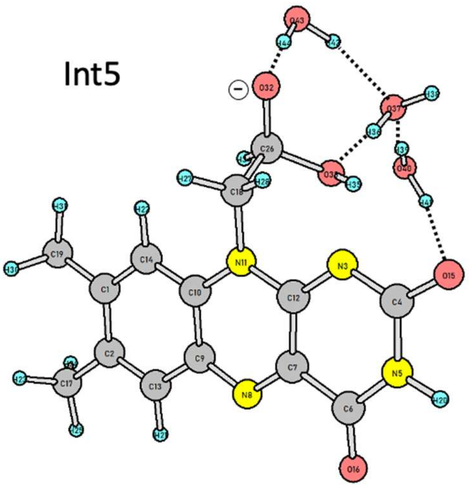

Fig. S5-3
$\Delta G^{\circ} = -3.67$ kcal/mol

TS9 in Figure S5-4 | TS10 in Figure S5-6

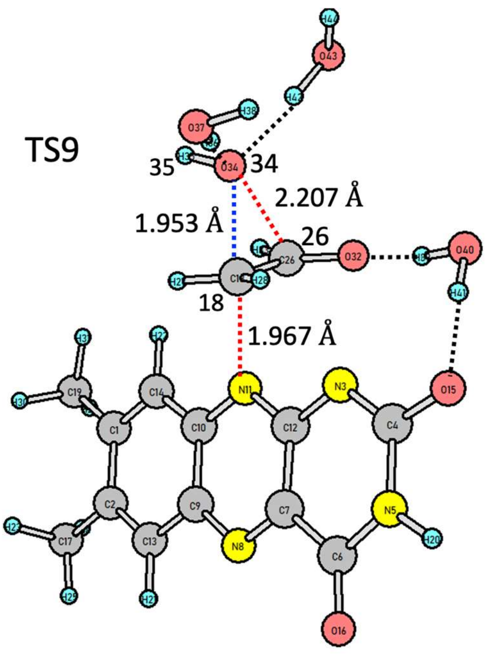

Fig. S5-4
$\Delta G^{\ddagger} = +29.99$ kcal/mol
$\nu^{\ddagger} = 664.2548i$ cm^{-1}

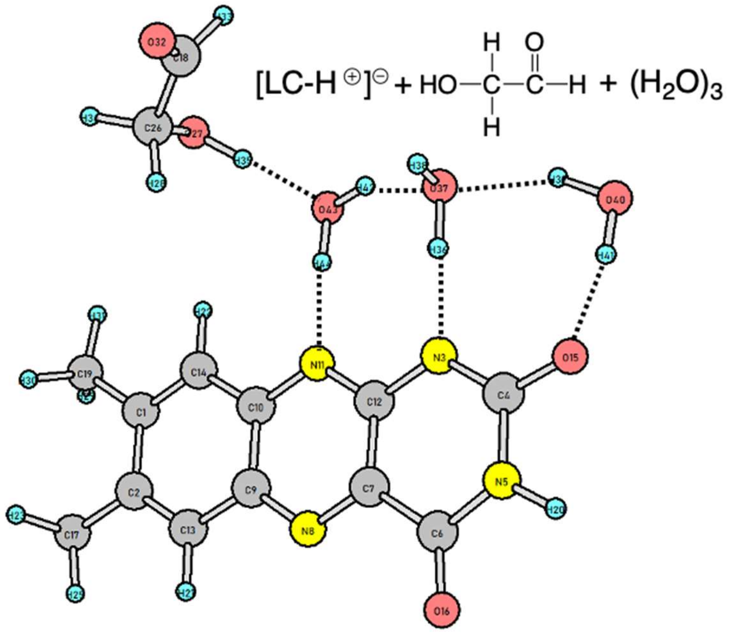

Fig. S5-5
$\Delta G^{\circ} = -19.68$ kcal/mol

Int5 in Figure S5-3

↓

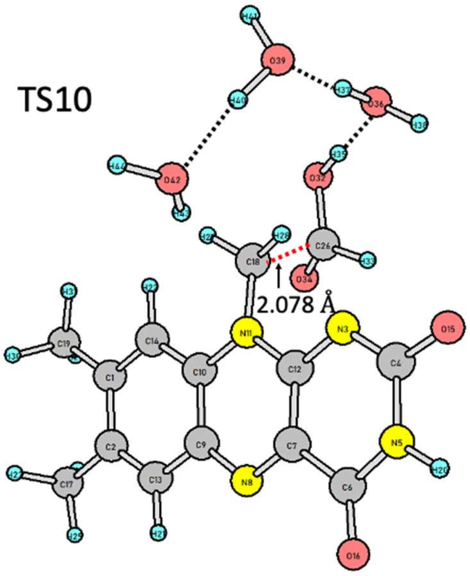

Fig. S5-6
$\Delta G^{\ddagger} = +17.18$ kcal/mol
$\nu^{\ddagger} = 569.8844i$ cm^{-1}

↓

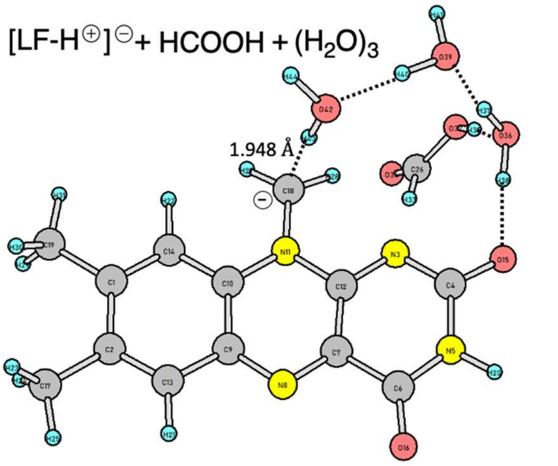

Fig. S5-7
$\Delta G^{\circ} = -0.49$ kcal/mol

↓

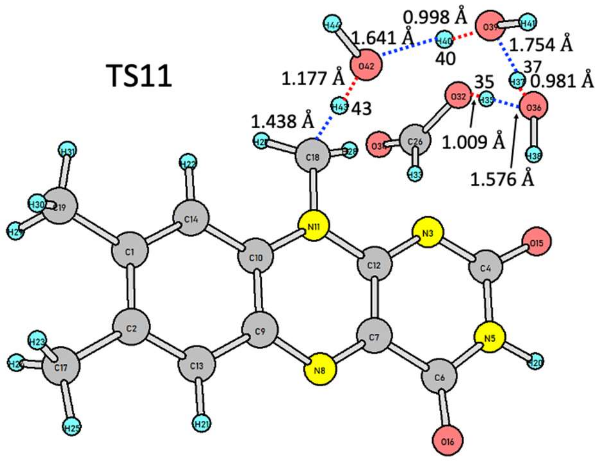

Fig. S5-8
$\Delta G^{\ddagger} = +2.70$ kcal/mol
$\nu^{\ddagger} = 1323.9417i$ cm^{-1}

↓

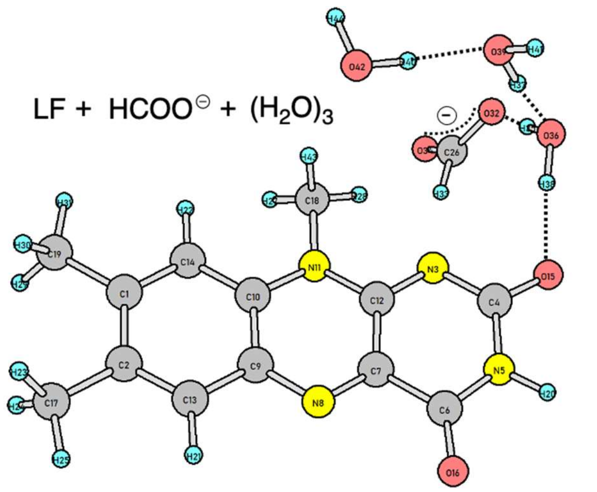

Fig. S5-9
ΔG° = -28.43 kcal/mol

Figure S5. Geometries of species in Scheme 6.

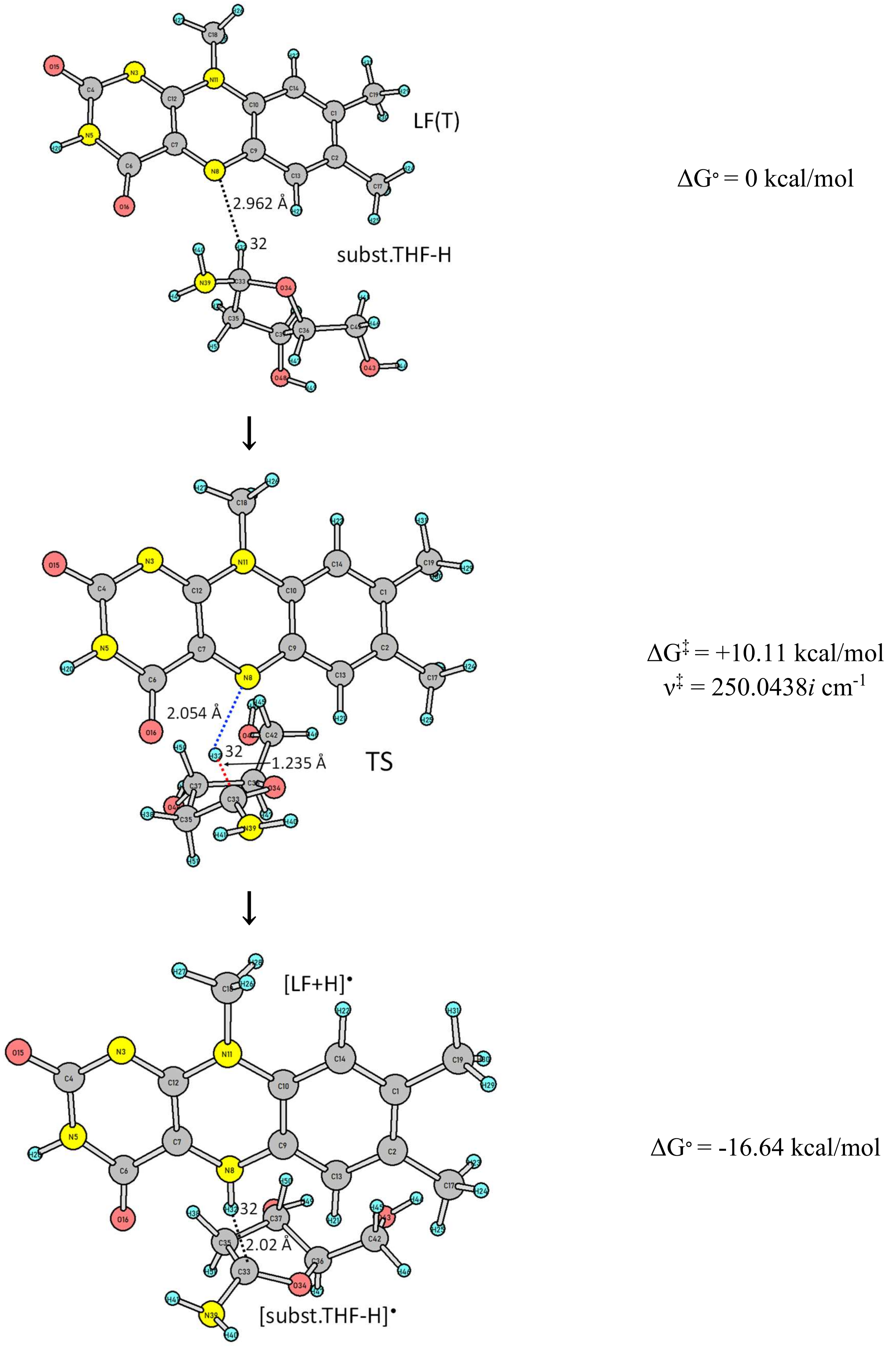

Figure S6. A hydrogen-shift reaction between the triplet lumiflavin, LF(T), and (*R*)-2-amino-(*S*)-4-hydroxy-(*R*)-5-(hydroxymethyl)tetrahydrofuran, "subst.THF". The migration of the α-position hydrogen H(32) of subst.THF is based on the result of calculated thermochemical data in Figure 2 of the reference (Martin, et al., 2002).

An input data, Cartesian coordinates and energies obtained by wb97x-D/6-311+G(d,p) SCRF=(PCM, solvent=water) calculations

```
========An input data for DFT calculations========

%mem=500MW
%nproc=4
#  uwb97xd    /  6-311+G**    6d
      pop=none  optcyc=300
   scf=(direct,pass)  scfcyc=300 iop(1/8=2)
   scrf=(pcm,solvent=water)
   opt=noeig    freq=noraman

  vitamin B2    C17H20N4O6  triplet radical
  vita-b20t.log----title------

   0    3
  (hereafter, XYZ coodinates shown below)

   ==================Figure S1=================

   The reaction from riboflavin(RF) to
 7,8-dimethyl-10-formylmethylflavin(FMF) in the
 triplet spin state.

    ---------Figure S1-1, reactant RF-----------

   vita-b20t.high.log

  Stoichiometry    C17H20N4O6(3)

                     Standard orientation:
```

Center Number	Atomic Number	Atomic Type	X	Y	Z
1	6	0	1.047922	3.441297	-0.070658
2	6	0	2.450941	3.447300	0.220993
3	7	0	0.540605	-2.454799	-0.412531
4	6	0	1.152506	-3.672920	-0.157678
5	7	0	2.502595	-3.657261	0.118121
6	6	0	3.312080	-2.534901	0.226856
7	6	0	2.618866	-1.268473	-0.010514
8	7	0	3.299608	-0.119186	0.128101
9	6	0	2.550404	1.017123	0.036220
10	6	0	1.143708	1.025410	-0.306364
11	7	0	0.551427	-0.162795	-0.625065
12	6	0	1.249158	-1.355250	-0.350687
13	6	0	3.154163	2.247658	0.268274
14	6	0	0.430511	2.243699	-0.330958
15	8	0	0.521489	-4.713933	-0.183399
16	8	0	4.494955	-2.645691	0.497363
17	6	0	3.154806	4.738214	0.479410
18	6	0	-0.784833	-0.228803	-1.223187
19	6	0	0.268229	4.722567	-0.084486
20	1	0	2.941757	-4.552070	0.291036
21	1	0	4.212702	2.248378	0.499014
22	1	0	-0.631907	2.238480	-0.539687
23	1	0	3.083054	5.392844	-0.394994
24	1	0	2.684997	5.273264	1.310214
25	1	0	4.206784	4.580598	0.712565
26	1	0	-0.792622	-1.046363	-1.943435
27	6	0	-1.914383	-0.430681	-0.203947
28	1	0	-0.938314	0.694663	-1.775642
29	1	0	0.302749	5.212353	0.892943
30	1	0	0.681764	5.426435	-0.812227
31	1	0	-0.775714	4.540532	-0.338954
32	8	0	-2.172670	-1.801404	0.040412
33	1	0	-1.328047	-2.278357	-0.036041
34	6	0	-3.198366	0.239462	-0.721521
35	1	0	-1.632132	0.064262	0.734479
36	6	0	-4.487997	-0.285334	-0.066117
37	8	0	-3.051247	1.646540	-0.639216
38	1	0	-3.077811	1.914847	0.283463
39	8	0	-5.627451	0.337167	-0.637655
40	1	0	-5.705389	0.065155	-1.554517
41	1	0	-4.517608	-1.365143	-0.248990

42	6	0	-4.618474	-0.050847	1.438947
43	8	0	-3.590825	-0.651045	2.205236
44	1	0	-3.242921	-1.413578	1.725825
45	1	0	-4.609959	1.022174	1.658348
46	1	0	-5.600027	-0.433754	1.739489
47	1	0	-3.290447	0.027366	-1.792710

Standard basis: 6-311+G(d,p) (6D, 7F)

SCF Done: E(UwB97XD) = -1330.15560294 A.U. after 1 cycles

Zero-point correction= 0.373246 (a.u.)
Thermal correction to Energy= 0.398625
Thermal correction to Enthalpy= 0.399569
Thermal correction to Gibbs Free Energy= 0.316615
Sum of electronic and zero-point Energies= -1329.782357
Sum of electronic and thermal Energies= -1329.756978
Sum of electronic and thermal Enthalpies= -1329.756034
Sum of electronic and thermal Free Energies= -1329.838988

	E (Thermal)	CV	S
	KCal/Mol	Cal/Mol-Kelvin	Cal/Mol-Kelvin
Total	250.141	97.153	174.591

Item	Value	Threshold	Converged?
Maximum Force	0.000008	0.000450	YES
RMS Force	0.000002	0.000300	YES
Maximum Displacement	0.001411	0.001800	YES
RMS Displacement	0.000246	0.001200	YES

--------Figure S1-2, TS1(T)----------

H(33) migration from O(33) to N(3)

vita-b20t-ts.high.log

Stoichiometry C17H20N4O6(3)

Standard orientation:

Center Number	Atomic Number	Atomic Type	Coordinates (Angstroms) X	Y	Z
1	6	0	-2.268497	-3.068904	0.043954
2	6	0	-3.584080	-2.583963	0.268221
3	7	0	0.293976	2.325438	-0.409768
4	6	0	0.167490	3.689262	-0.159446
5	7	0	-1.123239	4.130630	0.029817
6	6	0	-2.284486	3.353293	0.125719
7	6	0	-2.068399	1.913326	-0.059097
8	7	0	-3.114626	1.071169	0.059184
9	6	0	-2.810620	-0.266823	0.019575
10	6	0	-1.498052	-0.773923	-0.255607
11	7	0	-0.505332	0.141854	-0.586453
12	6	0	-0.765888	1.495229	-0.346163
13	6	0	-3.820693	-1.201633	0.252711
14	6	0	-1.253684	-2.156956	-0.212780
15	8	0	1.138618	4.426554	-0.130330
16	8	0	-3.362020	3.886471	0.341700
17	6	0	-4.711961	-3.535155	0.532349
18	6	0	0.769106	-0.248244	-1.189372
19	6	0	-1.971702	-4.543393	0.090477
20	1	0	-1.236441	5.126513	0.180231
21	1	0	-4.820047	-0.822622	0.430442
22	1	0	-0.249159	-2.529696	-0.362433
23	1	0	-4.832193	-4.236425	-0.300023
24	1	0	-4.509547	-4.141790	1.421319
25	1	0	-5.654425	-3.009094	0.680749
26	1	0	0.992543	0.442222	-2.004743
27	6	0	1.945113	-0.217340	-0.176808
28	1	0	0.662478	-1.243689	-1.606738
29	1	0	-2.229580	-4.967200	1.065719
30	1	0	-2.556978	-5.086800	-0.657518
31	1	0	-0.915784	-4.739426	-0.097054
32	8	0	2.353985	1.065778	0.102799
33	1	0	1.339256	1.874843	-0.257140
34	6	0	3.117775	-1.053016	-0.814776
35	1	0	1.642185	-0.758291	0.735626
36	6	0	4.446629	-0.818858	-0.057724

37	8	0	2.737261	-2.412605	-0.898268
38	1	0	2.850117	-2.839583	-0.039436
39	8	0	5.433561	-1.754557	-0.490287
40	1	0	5.458279	-1.769001	-1.454569
41	1	0	4.759126	0.208130	-0.282737
42	6	0	4.365210	-0.956115	1.471836
43	8	0	3.968891	0.243714	2.126391
44	1	0	3.333603	0.718879	1.551536
45	1	0	3.699551	-1.786433	1.751817
46	1	0	5.364333	-1.210439	1.829636
47	1	0	3.245314	-0.717283	-1.848584

Standard basis: 6-311+G(d,p) (6D, 7F)

SCF Done: E(UwB97XD) = -1330.12654132 A.U. after 8 cycles
<Sx>= 0.0000 <Sy>= 0.0000 <Sz>= 1.0000

Harmonic frequencies (cm**-1), IR intensities (KM/Mole), Raman

	1	2	3
	A	A	A
Frequencies --	-1376.9802	25.4023	32.6509

Zero-point correction= 0.363153 (a.u.)
Thermal correction to Energy= 0.388034
Thermal correction to Enthalpy= 0.388979
Thermal correction to Gibbs Free Energy= 0.307509
Sum of electronic and zero-point Energies= -1329.763388
Sum of electronic and thermal Energies= -1329.738507
Sum of electronic and thermal Enthalpies= -1329.737562
Sum of electronic and thermal Free Energies= -1329.819032

	E (Thermal)	CV	S
	KCal/Mol	Cal/Mol-Kelvin	Cal/Mol-Kelvin
Total	243.495	96.167	171.467

Item	Value	Threshold	Converged?
Maximum Force	0.000007	0.000450	YES
RMS Force	0.000001	0.000300	YES
Maximum Displacement	0.000996	0.001800	YES
RMS Displacement	0.000142	0.001200	YES

------- Figure S1-3, Int1(T)---------

vita-b20t-for2a.high.log

Stoichiometry C17H20N4O6(3)

Standard orientation:

Center Number	Atomic Number	Atomic Type	Coordinates (Angstroms) X	Y	Z
1	6	0	-1.636059	3.305361	-0.046703
2	6	0	-3.016388	3.094946	-0.258000
3	7	0	-0.154597	-2.518650	0.282361
4	6	0	-0.568516	-3.822370	0.123765
5	7	0	-1.923724	-3.969342	-0.030291
6	6	0	-2.879307	-2.959509	-0.149982
7	6	0	-2.358288	-1.601147	-0.018729
8	7	0	-3.226792	-0.564949	-0.116322
9	6	0	-2.675578	0.678772	-0.067061
10	6	0	-1.300706	0.911342	0.172040
11	7	0	-0.473858	-0.203640	0.423842
12	6	0	-1.001886	-1.442423	0.220415
13	6	0	-3.502175	1.802007	-0.266803
14	6	0	-0.803688	2.210836	0.162740
15	8	0	0.209753	-4.755975	0.122851
16	8	0	-4.046940	-3.253680	-0.331440
17	6	0	-3.941255	4.261177	-0.479528
18	6	0	0.825697	-0.030252	1.074886
19	6	0	-1.062440	4.695359	-0.051200
20	1	0	-2.257869	-4.918352	-0.132488
21	1	0	-4.556670	1.612559	-0.437616
22	1	0	0.252992	2.395881	0.310221
23	1	0	-3.922238	4.947444	0.372281
24	1	0	-3.645688	4.837580	-1.361236
25	1	0	-4.968816	3.924641	-0.622379
26	1	0	0.971878	-0.843505	1.789002
27	6	0	1.985874	-0.011646	0.074805

28	1	0	0.792131	0.890904	1.649946
29	1	0	-1.249289	5.196347	-1.005891
30	1	0	-1.520227	5.313035	0.727527
31	1	0	0.014886	4.679270	0.118712
32	8	0	2.206334	-1.170244	-0.517761
33	1	0	0.848902	-2.360050	0.185604
34	6	0	3.408943	0.344445	0.894847
35	1	0	1.924718	0.832292	-0.635095
36	6	0	4.447785	0.924670	-0.065499
37	8	0	3.128444	1.229934	1.906548
38	1	0	3.178547	2.119703	1.525801
39	8	0	4.008764	2.247096	-0.342320
40	1	0	4.762325	2.808036	-0.535853
41	1	0	5.385222	0.948141	0.502564
42	6	0	4.666034	0.154188	-1.368446
43	8	0	4.963522	-1.208414	-1.146048
44	1	0	4.120056	-1.662323	-1.044140
45	1	0	3.788385	0.273154	-2.011652
46	1	0	5.519562	0.598544	-1.884528
47	1	0	3.726690	-0.601501	1.328168

SCF Done: E(UwB97XD) = -1330.15316419 A.U. after 1 cycles
NFock= 1 Conv=0.48D-08 -V/T= 2.0047
<Sx>= 0.0000 <Sy>= 0.0000 <Sz>= 1.0000

Zero-point correction= 0.373317 (a.u.)
Thermal correction to Energy= 0.398490
Thermal correction to Enthalpy= 0.399435
Thermal correction to Gibbs Free Energy= 0.316957
Sum of electronic and zero-point Energies= -1329.779847
Sum of electronic and thermal Energies= -1329.754674
Sum of electronic and thermal Enthalpies= -1329.753730
Sum of electronic and thermal Free Energies= -1329.836207

	E (Thermal) KCal/Mol	CV Cal/Mol-Kelvin	S Cal/Mol-Kelvin
Total	250.057	96.833	173.588

Item	Value	Threshold	Converged?
Maximum Force	0.000084	0.000450	YES
RMS Force	0.000008	0.000300	YES
Maximum Displacement	0.000377	0.001800	YES
RMS Displacement	0.000073	0.001200	YES

-------Figure S1-4, TS2(T), C(27)....C(34) cleavage---------

vita-b20t-ts2a.high.log

Stoichiometry C17H20N4O6(3)

Standard orientation:

Center Number	Atomic Number	Atomic Type	Coordinates (Angstroms) X	Y	Z
1	6	0	0.973089	3.064147	0.321866
2	6	0	-0.058577	3.898532	-0.157732
3	7	0	-2.354224	-1.982550	0.452450
4	6	0	-3.570333	-2.570836	0.164806
5	7	0	-4.518980	-1.698388	-0.295642
6	6	0	-4.375741	-0.328447	-0.516907
7	6	0	-3.056571	0.214595	-0.198082
8	7	0	-2.851540	1.539161	-0.394437
9	6	0	-1.597182	1.996203	-0.135284
10	6	0	-0.553138	1.177882	0.358333
11	7	0	-0.850466	-0.179081	0.628173
12	6	0	-2.087572	-0.646278	0.291642
13	6	0	-1.308103	3.352989	-0.376415
14	6	0	0.711884	1.718109	0.563822
15	8	0	-3.760453	-3.760086	0.314443
16	8	0	-5.316713	0.314492	-0.942212
17	6	0	0.199468	5.355493	-0.430830
18	6	0	0.136148	-1.057521	1.235997
19	6	0	2.351808	3.613275	0.564114
20	1	0	-5.422075	-2.098445	-0.513085
21	1	0	-2.119160	3.967868	-0.751707
22	1	0	1.534519	1.104809	0.908558
23	1	0	0.473662	5.885664	0.486234
24	1	0	1.026333	5.484813	-1.134995

25	1	0	-0.684336	5.838001	-0.849697
26	1	0	-0.326399	-1.730606	1.958898
27	6	0	0.909561	-1.841225	0.191440
28	1	0	0.843010	-0.457867	1.810554
29	1	0	2.834937	3.894950	-0.377074
30	1	0	2.315793	4.511714	1.186180
31	1	0	2.983589	2.881722	1.067704
32	8	0	1.204354	-3.003302	0.341314
33	1	0	-1.651968	-2.634668	0.767282
34	6	0	3.697175	-0.952903	0.712472
35	1	0	1.152160	-1.289852	-0.732577
36	6	0	4.568515	-1.258611	-0.468453
37	8	0	3.811320	0.294940	1.232565
38	1	0	4.177206	0.848754	0.525556
39	8	0	4.644504	-0.052460	-1.226765
40	1	0	5.498666	-0.000630	-1.659430
41	1	0	5.576323	-1.527418	-0.112281
42	6	0	4.041291	-2.400021	-1.344823
43	8	0	3.865604	-3.599712	-0.618145
44	1	0	2.964355	-3.607838	-0.273447
45	1	0	3.109431	-2.079257	-1.823248
46	1	0	4.770579	-2.592976	-2.135708
47	1	0	3.490572	-1.716002	1.453073

Standard basis: 6-311+G(d,p) (6D, 7F)

SCF Done: E(UwB97XD) = -1330.14403494 A.U. after 1 cycles
NFock= 1 Conv=0.35D-08 -V/T= 2.0047

Harmonic frequencies (cm**-1), IR intensities (KM/Mole), Raman

	1	2	3
	A	A	A
Frequencies --	-20.5900	14.8521	20.9328

Zero-point correction= 0.369869 (a.u.)
Thermal correction to Energy= 0.395952
Thermal correction to Enthalpy= 0.396896
Thermal correction to Gibbs Free Energy= 0.311250
Sum of electronic and zero-point Energies= -1329.774165
Sum of electronic and thermal Energies= -1329.748083
Sum of electronic and thermal Enthalpies= -1329.747139
Sum of electronic and thermal Free Energies= -1329.832785

	E (Thermal)	CV	S
	KCal/Mol	Cal/Mol-Kelvin	Cal/Mol-Kelvin
Total	248.464	98.041	180.258

Item	Value	Threshold	Converged?
Maximum Force	0.000019	0.000450	YES
RMS Force	0.000003	0.000300	YES
Maximum Displacement	0.002605	0.001800	NO
RMS Displacement	0.000653	0.001200	YES

-----------Figure S1-5, Int2(T)----------

vita-b2.test11a.high.log

Stoichiometry C17H20N4O6(3)

Standard orientation:

Center Number	Atomic Number	Atomic Type	Coordinates (Angstroms) X	Y	Z
1	6	0	-3.942747	-2.079350	-0.147611
2	6	0	-4.864997	-1.022616	-0.303231
3	7	0	0.953859	1.446694	0.166236
4	6	0	1.438541	2.730162	0.240163
5	7	0	0.490650	3.705138	0.161200
6	6	0	-0.886313	3.530139	-0.012656
7	6	0	-1.326581	2.138310	-0.086005
8	7	0	-2.650476	1.895056	-0.236686
9	6	0	-3.023274	0.586984	-0.230463
10	6	0	-2.109646	-0.487407	-0.104264
11	7	0	-0.731307	-0.175773	-0.033930
12	6	0	-0.372804	1.139541	0.012097
13	6	0	-4.391951	0.274084	-0.337478
14	6	0	-2.583964	-1.793858	-0.049557
15	8	0	2.633463	2.959689	0.367207

16	8	0	-1.610705	4.503432	-0.081930
17	6	0	-6.338256	-1.304110	-0.418657
18	6	0	0.266882	-1.220641	0.111720
19	6	0	-4.415394	-3.505035	-0.084196
20	1	0	0.822493	4.659283	0.215477
21	1	0	-5.077953	1.107873	-0.442131
22	1	0	-1.908066	-2.628097	0.082142
23	1	0	-6.550974	-1.942916	-1.280862
24	1	0	-6.710220	-1.827363	0.467125
25	1	0	-6.905991	-0.379905	-0.531396
26	6	0	0.581085	-1.506601	1.570388
27	1	0	-0.098527	-2.139201	-0.348032
28	1	0	1.178114	-0.982901	-0.439692
29	1	0	-5.106696	-3.653409	0.750855
30	1	0	-4.953157	-3.781736	-0.996034
31	1	0	-3.579852	-4.194814	0.039655
32	8	0	1.636559	-1.956974	1.934123
33	1	0	1.677418	0.734068	0.154576
34	1	0	3.076611	-2.596973	0.853735
35	1	0	-0.223586	-1.275507	2.289774
36	8	0	3.687323	-3.126730	0.324087
37	6	0	4.129412	-2.376598	-0.787032
38	6	0	5.208587	-1.351734	-0.416545
39	1	0	3.299564	-1.870177	-1.295031
40	1	0	4.560478	-3.089475	-1.494555
41	6	0	4.705652	-0.203277	0.378619
42	8	0	5.804223	-0.835341	-1.622796
43	1	0	5.971120	-1.859228	0.185377
44	1	0	6.303664	-1.542497	-2.039400
45	1	0	5.367918	0.356220	1.030031
46	8	0	3.643369	0.471364	-0.142358
47	1	0	3.619323	1.397473	0.162220

Standard basis: 6-311+G(d,p) (6D, 7F)

```
Zero-point correction=                           0.369497 (a.u.)
Thermal correction to Energy=                    0.396392
Thermal correction to Enthalpy=                  0.397336
Thermal correction to Gibbs Free Energy=         0.310078
Sum of electronic and zero-point Energies=       -1329.778632
Sum of electronic and thermal Energies=          -1329.751737
Sum of electronic and thermal Enthalpies=        -1329.750793
Sum of electronic and thermal Free Energies=     -1329.838051
```

	E (Thermal) KCal/Mol	CV Cal/Mol-Kelvin	S Cal/Mol-Kelvin
Total	248.740	99.948	183.650

Item	Value	Threshold	Converged?
Maximum Force	0.000024	0.000450	YES
RMS Force	0.000004	0.000300	YES
Maximum Displacement	0.000919	0.001800	YES
RMS Displacement	0.000214	0.001200	YES

------Figure S1-6, TS3(T), N(3)....H(33)....C(34)---------

vita-b2.test33n.high.log

Stoichiometry C17H20N4O6(3)

Standard orientation:

Center Number	Atomic Number	Atomic Type	Coordinates (Angstroms) X	Y	Z
1	6	0	-4.263313	-1.115130	0.060561
2	6	0	-4.678401	0.163719	0.509161
3	7	0	1.445670	0.395179	-1.041264
4	6	0	2.341371	1.459576	-1.151666
5	7	0	1.879322	2.698154	-0.774250
6	6	0	0.609531	3.009206	-0.294737
7	6	0	-0.325993	1.887747	-0.276806
8	7	0	-1.612536	2.121213	0.104502
9	6	0	-2.436218	1.047627	0.078707
10	6	0	-2.039494	-0.241807	-0.372302
11	7	0	-0.719602	-0.404090	-0.811048
12	6	0	0.145678	0.658821	-0.705116
13	6	0	-3.771687	1.201110	0.510404
14	6	0	-2.956250	-1.293200	-0.368180

15	8	0	3.476266	1.290709	-1.551323
16	8	0	0.348389	4.151950	0.037303
17	6	0	-6.090623	0.381822	0.976805
18	6	0	-0.294967	-1.616877	-1.500336
19	6	0	-5.223319	-2.271270	0.051918
20	1	0	2.530689	3.467920	-0.856372
21	1	0	-4.066137	2.188467	0.849189
22	1	0	-2.661453	-2.281688	-0.695452
23	1	0	-6.806815	0.156765	0.181051
24	1	0	-6.331634	-0.273881	1.818597
25	1	0	-6.244263	1.414076	1.292701
26	6	0	0.170487	-2.688960	-0.537748
27	1	0	-1.133122	-2.007437	-2.083283
28	1	0	0.514870	-1.377219	-2.184305
29	1	0	-5.603334	-2.475279	1.057460
30	1	0	-6.090952	-2.057310	-0.579457
31	1	0	-4.746586	-3.178104	-0.321771
32	8	0	1.138808	-3.375410	-0.747391
33	1	0	1.966225	-0.329487	0.113523
34	6	0	2.203386	-0.703251	1.281288
35	1	0	1.659976	-1.646474	1.346327
36	8	0	1.657420	0.213791	2.126155
37	1	0	2.329763	0.896562	2.277596
38	1	0	-0.422691	-2.809476	0.387415
39	6	0	3.711576	-0.843136	1.389414
40	6	0	4.328691	-1.585838	0.201981
41	8	0	3.926072	-2.942500	0.174379
42	1	0	4.082122	-1.064007	-0.727893
43	1	0	5.414227	-1.571162	0.321012
44	1	0	3.098097	-3.033206	-0.307981
45	8	0	4.206491	0.479300	1.504516
46	1	0	3.917142	-1.411417	2.306851
47	1	0	5.033607	0.475483	1.990011

```
---------------------------------------------------------------------

Standard basis: 6-311+G(d,p) (6D, 7F)

SCF Done:  E(UwB97XD) =  -1330.11127647 A.U. after 2 cycles
         NFock=  2  Conv=0.14D-08     -V/T= 2.0047

Harmonic frequencies (cm**-1), IR intensities (KM/Mole), Raman
                 1                   2                   3
                 A                   A                   A
Frequencies --  -1212.2537             18.5999             36.8399

Zero-point correction=                         0.364878 (a.u.)
Thermal correction to Energy=                   0.390928
Thermal correction to Enthalpy=                 0.391873
Thermal correction to Gibbs Free Energy=        0.307146
Sum of electronic and zero-point Energies=        -1329.746398
Sum of electronic and thermal Energies=           -1329.720348
Sum of electronic and thermal Enthalpies=         -1329.719404
Sum of electronic and thermal Free Energies=      -1329.804131

                E (Thermal)          CV              S
                 KCal/Mol      Cal/Mol-Kelvin   Cal/Mol-Kelvin
Total              245.311           97.704          178.322

       Item             Value     Threshold  Converged?
Maximum Force           0.000019    0.000450    YES
RMS     Force          0.000004    0.000300    YES
Maximum Displacement     0.002240    0.001800    NO
RMS    Displacement    0.000507    0.001200    YES

  ------- Figure S1-7, FMF(T) + glecerol---------

   vita-b2.test33n.rev.high.log

Stoichiometry   C17H20N4O6(3)

                    Standard orientation:
---------------------------------------------------------------------
```

Center Number	Atomic Number	Atomic Type	X	Y	Z
1	6	0	-4.322580	-1.714770	-0.147732
2	6	0	-5.058604	-0.495558	-0.331912
3	7	0	0.981277	0.897451	0.469360
4	6	0	1.697457	2.083331	0.378952
5	7	0	0.988140	3.229029	0.087097

(Coordinates (Angstroms) span X, Y, Z.)

6	6	0	-0.384006	3.328142	-0.097473
7	6	0	-1.099355	2.058023	0.016257
8	7	0	-2.437190	2.043576	-0.139240
9	6	0	-3.015751	0.817047	-0.098168
10	6	0	-2.290263	-0.416531	0.145144
11	7	0	-0.952864	-0.325942	0.433071
12	6	0	-0.313642	0.917533	0.297965
13	6	0	-4.393333	0.717190	-0.308374
14	6	0	-2.970373	-1.647643	0.083718
15	8	0	2.900873	2.111098	0.552772
16	8	0	-0.905342	4.404137	-0.332105
17	6	0	-6.534021	-0.542114	-0.560836
18	6	0	-0.124134	-1.489690	0.723293
19	6	0	-5.023225	-3.038361	-0.212665
20	1	0	1.518428	4.088263	0.023498
21	1	0	-4.936364	1.640864	-0.468129
22	1	0	-2.427418	-2.574303	0.205370
23	1	0	-6.766776	-1.135164	-1.450427
24	1	0	-7.037055	-1.027804	0.280958
25	1	0	-6.948860	0.456812	-0.687576
26	6	0	0.885667	-1.248442	1.825855
27	1	0	-0.764283	-2.286060	1.103894
28	1	0	0.376615	-1.853688	-0.176523
29	1	0	-5.811046	-3.100924	0.543441
30	1	0	-5.500745	-3.180692	-1.186397
31	1	0	-4.325967	-3.859730	-0.049813
32	8	0	1.920842	-1.867649	1.886222
33	1	0	4.184630	-0.038979	0.628496
34	6	0	4.835624	-0.878090	0.351208
35	1	0	4.631404	-1.715096	1.020603
36	8	0	6.201809	-0.534889	0.523113
37	1	0	6.408306	0.110147	-0.159526
38	1	0	0.578208	-0.565854	2.633488
39	6	0	4.538061	-1.273408	-1.083457
40	6	0	3.072734	-1.656378	-1.291199
41	8	0	2.733174	-2.872981	-0.650034
42	1	0	2.435238	-0.831490	-0.945751
43	1	0	2.894093	-1.794827	-2.360588
44	1	0	2.545158	-2.684411	0.280478
45	8	0	4.863046	-0.133204	-1.877531
46	1	0	5.176591	-2.120163	-1.364551
47	1	0	4.997569	-0.409481	-2.785900

Standard basis: 6-311+G(d,p) (6D, 7F)

SCF Done: E(UwB97XD) = -1330.13679806 A.U. after 2 cycles
NFock= 2 Conv=0.17D-08 -V/T= 2.0047
<Sx>= 0.0000 <Sy>= 0.0000 <Sz>= 1.0000

Zero-point correction= 0.368798 (a.u.)
Thermal correction to Energy= 0.396019
Thermal correction to Enthalpy= 0.396964
Thermal correction to Gibbs Free Energy= 0.307669
Sum of electronic and zero-point Energies= -1329.768000
Sum of electronic and thermal Energies= -1329.740779
Sum of electronic and thermal Enthalpies= -1329.739834
Sum of electronic and thermal Free Energies= -1329.829129

	E (Thermal) KCal/Mol	CV Cal/Mol-Kelvin	S Cal/Mol-Kelvin
Total	248.506	99.868	187.936

Item	Value	Threshold	Converged?
Maximum Force	0.000034	0.000450	YES
RMS Force	0.000004	0.000300	YES
Maximum Displacement	0.008065	0.001800	NO
RMS Displacement	0.002058	0.001200	NO

-------glycerol, HO-CH2-CH(OH)-CH2-OH--------

glycerol.high.log

Stoichiometry C3H8O3

Standard orientation:

Center Number	Atomic Number	Atomic Type	Coordinates (Angstroms) X	Y	Z

1	1	0	0.801739	-0.724131	1.401471
2	6	0	0.812470	-0.801333	0.305112
3	1	0	0.366240	-1.756183	0.022063
4	8	0	2.140924	-0.807608	-0.183519
5	1	0	2.484585	0.081564	-0.055724
6	6	0	0.008257	0.347024	-0.277555
7	6	0	-1.435321	0.378056	0.226698
8	8	0	-2.202917	-0.714624	-0.252783
9	1	0	-1.441809	0.420248	1.322297
10	1	0	-1.915529	1.285896	-0.145437
11	1	0	-2.053911	-1.476761	0.309082
12	8	0	0.686449	1.536463	0.119181
13	1	0	0.006191	0.260783	-1.370907
14	1	0	0.444410	2.252252	-0.471401

```
Standard basis: 6-311+G(d,p) (6D, 7F)

SCF Done:  E(RwB97XD) =  -344.806341663  A.U. after  1 cycles
         NFock=  1  Conv=0.30D-08     -V/T= 2.0046

Zero-point correction=                           0.119613 (a.u.)
Thermal correction to Energy=                     0.127123
Thermal correction to Enthalpy=                   0.128067
Thermal correction to Gibbs Free Energy=          0.088366
Sum of electronic and zero-point Energies=           -344.686729
Sum of electronic and thermal Energies=              -344.679219
Sum of electronic and thermal Enthalpies=            -344.678275
Sum of electronic and thermal Free Energies=         -344.717975

                E (Thermal)          CV               S
                 KCal/Mol       Cal/Mol-Kelvin    Cal/Mol-Kelvin
Total              79.771           25.940            83.557

      Item             Value     Threshold  Converged?
Maximum Force          0.000023    0.000450    YES
RMS    Force          0.000006    0.000300    YES
Maximum Displacement    0.001819    0.001800    NO
RMS    Displacement    0.000476    0.001200    YES
```

==============FIgure S3=============

From FMF(T) to LC and H2C=C=O(T)

----------Figure S3-1, FMF(T)---------

fmf-t.high.log

Stoichiometry   C14H12N4O3(3)

Standard orientation:

Center Number	Atomic Number	Atomic Type	Coordinates (Angstroms) X	Y	Z
1	6	0	3.402400	0.286240	-0.176082
2	6	0	3.382559	-1.133822	0.033742
3	7	0	-2.513748	0.934923	-0.349586
4	6	0	-3.738146	0.307343	-0.170208
5	7	0	-3.732629	-1.050578	0.064158
6	6	0	-2.612769	-1.864191	0.154952
7	6	0	-1.342812	-1.163511	-0.022724
8	7	0	-0.197202	-1.871041	0.062955
9	6	0	0.943612	-1.147403	-0.037986
10	6	0	0.975358	0.281034	-0.276654
11	7	0	-0.214562	0.933651	-0.459816
12	6	0	-1.416716	0.228904	-0.272050
13	6	0	2.173713	-1.800724	0.097282
14	6	0	2.211053	0.953120	-0.326731
15	8	0	-4.780839	0.934058	-0.222042
16	8	0	-2.727280	-3.059049	0.368670
17	6	0	4.666913	-1.882950	0.188310
18	6	0	-0.267057	2.360186	-0.715600
19	6	0	4.705164	1.026841	-0.226328
20	1	0	-4.631695	-1.497129	0.188786
21	1	0	2.148168	-2.870741	0.265122
22	1	0	2.238810	2.023050	-0.479801
23	1	0	5.295286	-1.755900	-0.698429
24	1	0	5.238421	-1.496075	1.037369

25	1	0	4.490493	-2.946720	0.341996
26	6	0	-0.122987	3.193988	0.540279
27	1	0	0.493571	2.648467	-1.443451
28	1	0	-1.233882	2.603031	-1.157145
29	1	0	5.262849	0.901334	0.706239
30	1	0	5.339829	0.649154	-1.033205
31	1	0	4.544178	2.092570	-0.387708
32	8	0	-0.017467	2.733317	1.645140
33	1	0	-0.136055	4.283326	0.360882

Standard basis: 6-311+G(d,p) (6D, 7F)

SCF Done: E(UwB97XD) = -985.322845491 A.U. after 1 cycles
NFock= 1 Conv=0.34D-08 -V/T= 2.0047
<Sx>= 0.0000 <Sy>= 0.0000 <Sz>= 1.0000

Zero-point correction= 0.247359 (a.u.)
Thermal correction to Energy= 0.265559
Thermal correction to Enthalpy= 0.266503
Thermal correction to Gibbs Free Energy= 0.199358
Sum of electronic and zero-point Energies= -985.075487
Sum of electronic and thermal Energies= -985.057287
Sum of electronic and thermal Enthalpies= -985.056342
Sum of electronic and thermal Free Energies= -985.123488

	E (Thermal) KCal/Mol	CV Cal/Mol-Kelvin	S Cal/Mol-Kelvin
Total	166.641	68.957	141.320

Item	Value	Threshold	Converged?
Maximum Force	0.000024	0.000450	YES
RMS Force	0.000003	0.000300	YES
Maximum Displacement	0.001085	0.001800	YES
RMS Displacement	0.000236	0.001200	YES

--------Figure S3-2, TS4(T), C(26)....H(32).....N(3)--------

fmf-t-det.high.log

Stoichiometry C14H12N4O3(3)

Standard orientation:

Center Number	Atomic Number	Atomic Type	Coordinates (Angstroms) X	Y	Z
1	6	0	-3.524836	0.552211	-0.182230
2	6	0	-3.642705	-0.818434	0.140662
3	7	0	2.479446	0.618799	-0.474505
4	6	0	3.639950	-0.106363	-0.250068
5	7	0	3.473289	-1.463468	-0.095407
6	6	0	2.269760	-2.147454	0.063132
7	6	0	1.077223	-1.301960	0.014114
8	7	0	-0.132068	-1.870608	0.213777
9	6	0	-1.206203	-1.038992	0.111248
10	6	0	-1.105581	0.348225	-0.163810
11	7	0	0.184221	0.900633	-0.285524
12	6	0	1.268604	0.050164	-0.246298
13	6	0	-2.496146	-1.577292	0.271061
14	6	0	-2.261566	1.108407	-0.334139
15	8	0	4.734072	0.423460	-0.231459
16	8	0	2.273610	-3.351226	0.245254
17	6	0	-4.997710	-1.445575	0.317960
18	6	0	0.376085	2.333056	-0.346825
19	6	0	-4.750251	1.402301	-0.371632
20	1	0	4.318064	-2.013459	-0.006946
21	1	0	-2.561443	-2.636350	0.495860
22	1	0	-2.199853	2.151222	-0.616700
23	1	0	-5.560130	-0.947601	1.113134
24	1	0	-5.592916	-1.360197	-0.596053
25	1	0	-4.910986	-2.502679	0.570876
26	6	0	1.585874	2.785052	0.482437
27	1	0	-0.504624	2.851719	0.019375
28	1	0	0.606165	2.665083	-1.366493
29	1	0	-5.387133	1.004303	-1.167217
30	1	0	-5.355619	1.426885	0.539520
31	1	0	-4.484570	2.428021	-0.629610
32	1	0	2.418320	1.934596	0.289010
33	8	0	1.715508	3.782879	1.087855

SCF Done: E(UwB97XD) = -985.290941237 A.U. after 1 cycles
NFock= 1 Conv=0.37D-08 -V/T= 2.0047
<Sx>= 0.0000 <Sy>= 0.0000 <Sz>= 1.0000

Harmonic frequencies (cm**-1), IR intensities (KM/Mole), Raman

	1	2	3
	A	A	A
Frequencies --	-1231.5711	35.8001	50.8384

Zero-point correction= 0.243237 (a.u.)
Thermal correction to Energy= 0.260844
Thermal correction to Enthalpy= 0.261788
Thermal correction to Gibbs Free Energy= 0.196395
Sum of electronic and zero-point Energies= -985.047705
Sum of electronic and thermal Energies= -985.030097
Sum of electronic and thermal Enthalpies= -985.029153
Sum of electronic and thermal Free Energies= -985.094546

	E (Thermal) KCal/Mol	CV Cal/Mol-Kelvin	S Cal/Mol-Kelvin
Total	163.682	67.814	137.631

Item	Value	Threshold	Converged?
Maximum Force	0.000054	0.000450	YES
RMS Force	0.000007	0.000300	YES
Maximum Displacement	0.001827	0.001800	NO
RMS Displacement	0.000453	0.001200	YES

------------Figure S3-3, Int3(T)--------------------

fmf-t-det.for.high.log

Stoichiometry C14H12N4O3(3)

Standard orientation:

Center Number	Atomic Number	Atomic Type	X	Y	Z
1	6	0	-3.429691	0.210146	-0.017871
2	6	0	-3.429679	-1.199446	0.049436
3	7	0	2.577710	0.814215	-0.100034
4	6	0	3.802176	0.185128	0.017001
5	7	0	3.726576	-1.180364	0.074339
6	6	0	2.573957	-1.966111	0.086722
7	6	0	1.318407	-1.224028	-0.024127
8	7	0	0.162337	-1.931075	-0.029537
9	6	0	-0.985162	-1.201095	-0.051708
10	6	0	-1.006519	0.209477	-0.147672
11	7	0	0.229184	0.882075	-0.283863
12	6	0	1.374715	0.156691	-0.130565
13	6	0	-2.221525	-1.869231	0.034021
14	6	0	-2.218456	0.890178	-0.115127
15	8	0	4.842509	0.807734	0.064534
16	8	0	2.670435	-3.175179	0.173956
17	6	0	-4.724561	-1.959373	0.146557
18	6	0	0.240835	2.281065	-0.651882
19	6	0	-4.721081	0.979048	0.015410
20	1	0	4.607403	-1.670831	0.155805
21	1	0	-2.191991	-2.951793	0.096151
22	1	0	-2.250354	1.971260	-0.157104
23	1	0	-5.279530	-1.678919	1.046730
24	1	0	-5.372425	-1.745593	-0.708543
25	1	0	-4.546134	-3.034752	0.178402
26	6	0	0.078864	3.233040	0.534499
27	1	0	-0.571242	2.479845	-1.355302
28	1	0	1.159192	2.543926	-1.180629
29	1	0	-5.362657	0.706134	-0.828034
30	1	0	-5.284883	0.762613	0.927694
31	1	0	-4.543569	2.054222	-0.027403
32	1	0	2.632605	1.819529	-0.046284
33	8	0	0.014463	4.404629	0.487013

Standard basis: 6-311+G(d,p) (6D, 7F)

SCF Done: E(UwB97XD) = -985.333788722 A.U. after 1 cycles
NFock= 1 Conv=0.33D-08 -V/T= 2.0047

<Sx>= 0.0000 <Sy>= 0.0000 <Sz>= 1.0000
<S**2>= 2.0297 S= 1.0099

Zero-point correction=	0.248271 (a.u.)
Thermal correction to Energy=	0.266646
Thermal correction to Enthalpy=	0.267590
Thermal correction to Gibbs Free Energy=	0.200319
Sum of electronic and zero-point Energies=	-985.085518
Sum of electronic and thermal Energies=	-985.067143
Sum of electronic and thermal Enthalpies=	-985.066199
Sum of electronic and thermal Free Energies=	-985.133470

	E (Thermal)	CV	S
	KCal/Mol	Cal/Mol-Kelvin	Cal/Mol-Kelvin
Total	167.323	69.888	141.583

Item	Value	Threshold	Converged?
Maximum Force	0.000021	0.000450	YES
RMS Force	0.000003	0.000300	YES
Maximum Displacement	0.001476	0.001800	YES
RMS Displacement	0.000282	0.001200	YES

--------Figure S3-4, TS5(T), N(11)....C(18) cleavage----------

fmf-t-ket.high.log

Stoichiometry C14H12N4O3(3)

Standard orientation:

Center Number	Atomic Number	Atomic Type	Coordinates (Angstroms) X	Y	Z
1	6	0	-3.343148	0.284898	-0.609473
2	6	0	-3.341748	-1.043229	-0.077023
3	7	0	2.571952	0.956842	-0.730409
4	6	0	3.804113	0.375663	-0.556504
5	7	0	3.766997	-0.904756	-0.033175
6	6	0	2.655015	-1.671386	0.272200
7	6	0	1.360001	-0.979788	0.037908
8	7	0	0.242764	-1.621808	0.310247
9	6	0	-0.913461	-0.976982	0.032241
10	6	0	-0.925493	0.344644	-0.476179
11	7	0	0.253361	1.045050	-0.631049
12	6	0	1.375400	0.335170	-0.457649
13	6	0	-2.146730	-1.642631	0.226458
14	6	0	-2.152086	0.948869	-0.795413
15	8	0	4.841551	0.933708	-0.838042
16	8	0	2.767032	-2.796093	0.701839
17	6	0	-4.640315	-1.769366	0.137770
18	6	0	0.246710	2.674565	0.423379
19	6	0	-4.642246	0.948557	-0.966057
20	1	0	4.669394	-1.340319	0.108744
21	1	0	-2.114262	-2.652582	0.619963
22	1	0	-2.149649	1.952343	-1.204696
23	1	0	-5.285022	-1.222577	0.831753
24	1	0	-5.193037	-1.872734	-0.800280
25	1	0	-4.467720	-2.765972	0.544614
26	6	0	0.109252	2.141761	1.761222
27	1	0	-0.620290	3.155619	-0.020300
28	1	0	1.205169	3.105126	0.168283
29	1	0	-5.181953	0.370195	-1.721488
30	1	0	-5.295815	1.020793	-0.091965
31	1	0	-4.477653	1.953187	-1.355668
32	1	0	2.566531	1.851107	-1.200246
33	8	0	-0.880182	1.899139	2.385541

Standard basis: 6-311+G(d,p) (6D, 7F)

SCF Done: E(UwB97XD) = -985.291419031 A.U. after 1 cycles
NFock= 1 Conv=0.26D-08 -V/T= 2.0047
<Sx>= 0.0000 <Sy>= 0.0000 <Sz>= 1.0000

Harmonic frequencies (cm**-1), IR intensities (KM/Mole), Raman

	1	2	3
	A	A	A
Frequencies --	-727.8287	38.0250	51.3680

Zero-point correction= 0.246496 (a.u.)

Thermal correction to Energy= 0.264487
Thermal correction to Enthalpy= 0.265431
Thermal correction to Gibbs Free Energy= 0.199139
Sum of electronic and zero-point Energies= -985.044923
Sum of electronic and thermal Energies= -985.026932
Sum of electronic and thermal Enthalpies= -985.025988
Sum of electronic and thermal Free Energies= -985.092280

	E (Thermal) KCal/Mol	CV Cal/Mol-Kelvin	S Cal/Mol-Kelvin
Total	165.968	68.740	139.524

Item	Value	Threshold	Converged?
Maximum Force	0.000013	0.000450	YES
RMS Force	0.000002	0.000300	YES
Maximum Displacement	0.000517	0.001800	YES
RMS Displacement	0.000092	0.001200	YES

-------Figure S3-5, LC + H2C=C=O(T)---------

fmf-t-ket.rev.high.log

Stoichiometry C14H12N4O3(3)

Standard orientation:

Center Number	Atomic Number	Atomic Type	Coordinates (Angstroms) X	Y	Z
1	6	0	-3.592723	1.337909	0.000192
2	6	0	-4.088834	-0.012115	-0.000976
3	7	0	2.172279	-0.047595	0.002399
4	6	0	3.120686	-1.030479	0.001931
5	7	0	2.622273	-2.325726	0.000771
6	6	0	1.300325	-2.732492	-0.000267
7	6	0	0.328211	-1.606945	0.000179
8	7	0	-0.953092	-1.873665	-0.000673
9	6	0	-1.803025	-0.827334	-0.000338
10	6	0	-1.313558	0.507805	0.000883
11	7	0	0.014621	0.767297	0.001802
12	6	0	0.810708	-0.270067	0.001459
13	6	0	-3.202322	-1.053566	-0.001225
14	6	0	-2.242031	1.573212	0.001099
15	8	0	4.314042	-0.806084	0.002492
16	8	0	1.000583	-3.904478	-0.001435
17	6	0	-5.570553	-0.265627	-0.001893
18	6	0	4.997401	2.479900	-0.008496
19	6	0	-4.562145	2.485358	0.000368
20	1	0	3.321886	-3.056817	0.000443
21	1	0	-3.547553	-2.081599	-0.002123
22	1	0	-1.854025	2.585672	0.001994
23	1	0	-6.045863	0.177559	0.877810
24	1	0	-6.045070	0.179191	-0.881198
25	1	0	-5.783078	-1.334930	-0.002971
26	6	0	3.609008	2.825944	0.000036
27	1	0	5.751968	3.262794	-0.013004
28	1	0	5.276733	1.434152	-0.010206
29	1	0	-5.211134	2.448059	-0.879225
30	1	0	-5.212327	2.446813	0.879021
31	1	0	-4.039142	3.441682	0.001396
32	1	0	2.505102	0.917957	0.003070
33	8	0	3.078255	3.894093	0.003591

Standard basis: 6-311+G(d,p) (6D, 7F)

SCF Done: E(UwB97XD) = -985.310976457 A.U. after 1 cycles
NFock= 1 Conv=0.33D-08 -V/T= 2.0047

Zero-point correction= 0.244438 (a.u.)
Thermal correction to Energy= 0.264244
Thermal correction to Enthalpy= 0.265188
Thermal correction to Gibbs Free Energy= 0.191749
Sum of electronic and zero-point Energies= -985.066539
Sum of electronic and thermal Energies= -985.046733
Sum of electronic and thermal Enthalpies= -985.045789
Sum of electronic and thermal Free Energies= -985.119227

E (Thermal)	CV	S
KCal/Mol	Cal/Mol-Kelvin	Cal/Mol-Kelvin

```
Total                165.815           72.163          154.565

      Item             Value     Threshold  Converged?
Maximum Force            0.000004     0.000450     YES
RMS     Force            0.000001     0.000300     YES
Maximum Displacement     0.002417     0.001800     NO
RMS     Displacement     0.000421     0.001200     YES

 ============Figure S4,  from FMF(T) to LF(T) + CO =======

   --------Figure S4-1,  FMF(T)-----------
   ***** This is the same as Figure S3-1******

  ---------Figure S4-2,  TS6(T), C(18)......C(26) cleavage--------

   fmf-t-me.high.log

 Stoichiometry    C14H12N4O3(3)

                    Standard orientation:
```

Center Number	Atomic Number	Atomic Type	X	Y	Z
1	6	0	3.455449	0.361400	-0.261047
2	6	0	3.471344	-1.017778	0.069046
3	7	0	-2.510348	0.868691	-0.562863
4	6	0	-3.686307	0.179356	-0.397204
5	7	0	-3.625134	-1.167010	-0.017958
6	6	0	-2.494977	-1.911278	0.199629
7	6	0	-1.234480	-1.135857	0.001452
8	7	0	-0.108063	-1.737846	0.170266
9	6	0	1.042440	-1.019830	0.001992
10	6	0	1.035322	0.344698	-0.335784
11	7	0	-0.203570	0.979060	-0.523265
12	6	0	-1.377956	0.258165	-0.364670
13	6	0	2.269847	-1.676229	0.193456
14	6	0	2.251969	1.018811	-0.459184
15	8	0	-4.774207	0.692966	-0.567496
16	8	0	-2.531043	-3.078344	0.519993
17	6	0	4.772540	-1.739052	0.279893
18	6	0	-0.302558	2.344363	-0.712400
19	6	0	4.745706	1.115954	-0.397905
20	1	0	-4.514456	-1.636259	0.097316
21	1	0	2.233894	-2.728853	0.449839
22	1	0	2.287688	2.068313	-0.714621
23	1	0	5.389092	-1.706572	-0.622834
24	1	0	5.352557	-1.276196	1.083069
25	1	0	4.601605	-2.783885	0.539560
26	6	0	-0.315336	3.155595	1.138315
27	1	0	-1.284791	2.672493	-1.016779
28	1	0	0.557014	2.838966	-1.136516
29	1	0	5.310113	1.087131	0.538511
30	1	0	5.378832	0.663829	-1.166445
31	1	0	4.572344	2.158636	-0.663117
32	1	0	0.786564	3.021447	1.246810
33	8	0	-1.112083	2.554225	1.818554

```
Standard basis: 6-311+G(d,p) (6D, 7F)

SCF Done:  E(UwB97XD) =  -985.261949697  A.U. after 1 cycles
         NFock=  1  Conv=0.26D-08     -V/T= 2.0047
<Sx>= 0.0000 <Sy>= 0.0000 <Sz>= 1.0000

Harmonic frequencies (cm**-1), IR intensities (KM/Mole), Raman
                 1                   2                   3
                 A                   A                   A
Frequencies --  -416.8362             33.4654             48.8478

Zero-point correction=                         0.244675 (a.u.)
Thermal correction to Energy=                   0.262827
Thermal correction to Enthalpy=                 0.263771
Thermal correction to Gibbs Free Energy=        0.196681
Sum of electronic and zero-point Energies=         -985.017275
Sum of electronic and thermal Energies=            -984.999123
Sum of electronic and thermal Enthalpies=          -984.998179
Sum of electronic and thermal Free Energies=       -985.065269

                E (Thermal)           CV              S
```

	KCal/Mol	Cal/Mol-Kelvin	Cal/Mol-Kelvin
Total	164.926	68.723	141.203

Item	Value	Threshold	Converged?
Maximum Force	0.000003	0.000450	YES
RMS Force	0.000001	0.000300	YES
Maximum Displacement	0.000674	0.001800	YES
RMS Displacement	0.000116	0.001200	YES

--------Figure S4-3, Int4(T)---------

fmf-t-co.rev.high.log

Stoichiometry C14H12N4O3(3)

Standard orientation:

Center Number	Atomic Number	Atomic Type	Coordinates (Angstroms) X	Y	Z
1	6	0	3.362520	0.577232	-0.400260
2	6	0	3.336887	-0.842831	-0.368833
3	7	0	-2.595602	1.308632	-0.325686
4	6	0	-3.784099	0.640727	-0.285405
5	7	0	-3.759495	-0.761323	-0.263262
6	6	0	-2.650769	-1.566426	-0.277519
7	6	0	-1.370924	-0.804489	-0.321094
8	7	0	-0.257985	-1.472695	-0.328330
9	6	0	0.904862	-0.762535	-0.356726
10	6	0	0.941725	0.642572	-0.383074
11	7	0	-0.279801	1.348589	-0.372512
12	6	0	-1.478838	0.623272	-0.341178
13	6	0	2.118387	-1.476818	-0.350376
14	6	0	2.179628	1.292342	-0.406500
15	8	0	-4.865580	1.204523	-0.267796
16	8	0	-2.724478	-2.777325	-0.254380
17	6	0	4.617205	-1.629471	-0.354769
18	6	0	-0.341810	2.691916	-0.374029
19	6	0	4.675721	1.305051	-0.427063
20	1	0	-4.660600	-1.220598	-0.232727
21	1	0	2.052893	-2.558697	-0.327095
22	1	0	2.244303	2.370626	-0.428208
23	1	0	5.226582	-1.404395	-1.234468
24	1	0	5.218665	-1.383076	0.524893
25	1	0	4.416831	-2.700974	-0.342334
26	1	0	-1.015208	-0.275270	3.518004
27	1	0	-1.321802	3.136375	-0.358935
28	1	0	0.564128	3.269094	-0.392152
29	1	0	5.282181	1.044284	0.444842
30	1	0	5.254102	1.027719	-1.313109
31	1	0	4.531963	2.385408	-0.435405
32	6	0	-0.209708	0.049985	2.814836
33	8	0	0.921486	0.185793	3.096822

Standard basis: 6-311+G(d,p) (6D, 7F)

SCF Done: E(UwB97XD) = -985.274685119 A.U. after 1 cycles
NFock= 1 Conv=0.22D-08 -V/T= 2.0047
<Sx>= 0.0000 <Sy>= 0.0000 <Sz>= 1.0000

Zero-point correction= 0.241040 (a.u.)
Thermal correction to Energy= 0.261692
Thermal correction to Enthalpy= 0.262636
Thermal correction to Gibbs Free Energy= 0.186223
Sum of electronic and zero-point Energies= -985.033645
Sum of electronic and thermal Energies= -985.012993
Sum of electronic and thermal Enthalpies= -985.012049
Sum of electronic and thermal Free Energies= -985.088462

	E (Thermal) KCal/Mol	CV Cal/Mol-Kelvin	S Cal/Mol-Kelvin
Total	164.214	73.697	160.825

Item	Value	Threshold	Converged?
Maximum Force	0.000004	0.000450	YES
RMS Force	0.000001	0.000300	YES
Maximum Displacement	0.003398	0.001800	NO
RMS Displacement	0.000473	0.001200	YES

----------Figure S4-4, TS7(T), C(18).....H(26).....C(32)-------

fmf-t-co.high.log

Stoichiometry C14H12N4O3(3)

Standard orientation:

Center Number	Atomic Number	Atomic Type	Coordinates (Angstroms) X	Y	Z
1	6	0	3.438723	0.256573	-0.118098
2	6	0	3.467317	-1.127932	0.140878
3	7	0	-2.528032	0.716414	-0.349107
4	6	0	-3.707152	0.070706	-0.086477
5	7	0	-3.644609	-1.294507	0.169552
6	6	0	-2.510041	-2.082464	0.227281
7	6	0	-1.262965	-1.343789	-0.016471
8	7	0	-0.104355	-1.993629	0.070325
9	6	0	1.031918	-1.226490	-0.043544
10	6	0	1.028043	0.149066	-0.341494
11	7	0	-0.222399	0.760820	-0.642075
12	6	0	-1.418774	0.021774	-0.311827
13	6	0	2.273770	-1.833699	0.176646
14	6	0	2.215836	0.872341	-0.357456
15	8	0	-4.788066	0.642784	-0.077416
16	8	0	-2.587376	-3.276034	0.465418
17	6	0	4.772505	-1.829892	0.390198
18	6	0	-0.336922	1.984479	-1.147426
19	6	0	4.706773	1.064038	-0.133796
20	1	0	-4.526031	-1.759038	0.344411
21	1	0	2.273177	-2.896340	0.393040
22	1	0	2.212042	1.938687	-0.544431
23	1	0	5.440691	-1.729532	-0.470121
24	1	0	5.291804	-1.397326	1.250310
25	1	0	4.618310	-2.891952	0.581877
26	1	0	-0.417843	2.872048	0.105552
27	1	0	-1.324490	2.270007	-1.485765
28	1	0	0.539648	2.439780	-1.587997
29	1	0	5.217754	1.011727	0.832146
30	1	0	5.404777	0.685517	-0.886229
31	1	0	4.505103	2.112595	-0.354841
32	6	0	-0.527913	3.724316	1.019454
33	8	0	-0.470292	4.861499	0.948746

Standard basis: 6-311+G(d,p) (6D, 7F)

SCF Done: E(UwB97XD) = -985.246892631 A.U. after 1 cycles
NFock= 1 Conv=0.30D-08 -V/T= 2.0047
<Sx>= 0.0000 <Sy>= 0.0000 <Sz>= 1.0000

Harmonic frequencies (cm**-1), IR intensities (KM/Mole), Raman

	1	2	3
	A	A	A
Frequencies --	-1950.9671	17.1511	29.8055

Zero-point correction= 0.239400 (a.u.)
Thermal correction to Energy= 0.258234
Thermal correction to Enthalpy= 0.259178
Thermal correction to Gibbs Free Energy= 0.189291
Sum of electronic and zero-point Energies= -985.007492
Sum of electronic and thermal Energies= -984.988659
Sum of electronic and thermal Enthalpies= -984.987715
Sum of electronic and thermal Free Energies= -985.057602

	E (Thermal) KCal/Mol	CV Cal/Mol-Kelvin	S Cal/Mol-Kelvin
Total	162.044	70.326	147.090

Item	Value	Threshold	Converged?
Maximum Force	0.000021	0.000450	YES
RMS Force	0.000003	0.000300	YES
Maximum Displacement	0.001552	0.001800	YES
RMS Displacement	0.000329	0.001200	YES

-------Figure S4-5, LF(T) + CO----------

fmf-t-co.for.high.log

Stoichiometry   C14H12N4O3(3)

Standard orientation:

Center Number	Atomic Number	Atomic Type	Coordinates (Angstroms) X	Y	Z
1	6	0	3.176963	0.586457	-0.564103
2	6	0	3.151630	-0.845607	-0.548282
3	7	0	-2.721717	1.282174	-0.106943
4	6	0	-3.946988	0.637748	-0.017474
5	7	0	-3.948453	-0.739332	-0.020296
6	6	0	-2.832981	-1.560205	-0.103483
7	6	0	-1.562686	-0.845512	-0.201044
8	7	0	-0.426811	-1.566309	-0.289991
9	6	0	0.716860	-0.841552	-0.379467
10	6	0	0.753246	0.603155	-0.385733
11	7	0	-0.422191	1.290723	-0.281928
12	6	0	-1.626928	0.570673	-0.191326
13	6	0	1.940965	-1.511619	-0.461968
14	6	0	1.989144	1.272812	-0.484786
15	8	0	-4.985464	1.269864	0.061191
16	8	0	-2.953941	-2.773766	-0.091831
17	6	0	4.431864	-1.615251	-0.610903
18	6	0	-0.428177	2.751542	-0.257260
19	6	0	4.481344	1.321290	-0.656840
20	1	0	-4.847856	-1.197242	0.047804
21	1	0	1.914156	-2.594735	-0.448696
22	1	0	2.010880	2.353746	-0.491164
23	1	0	4.999469	-1.352144	-1.508263
24	1	0	5.066036	-1.369469	0.246794
25	1	0	4.250900	-2.689317	-0.613322
26	1	0	0.157639	3.105235	0.592275
27	1	0	-1.453012	3.088610	-0.158004
28	1	0	-0.005626	3.136848	-1.186143
29	1	0	5.138760	1.058372	0.176999
30	1	0	5.012292	1.064511	-1.578159
31	1	0	4.324813	2.399797	-0.642273
32	6	0	2.637050	-0.080673	2.741539
33	8	0	1.536645	0.062084	2.928520

Standard basis: 6-311+G(d,p) (6D, 7F)

SCF Done: E(UwB97XD) = -985.320305828 A.U. after 1 cycles
NFock= 1 Conv=0.37D-08   -V/T= 2.0047
<Sx>= 0.0000 <Sy>= 0.0000 <Sz>= 1.0000
<S**2>= 2.0282 S= 1.0094
<L.S>= 0.000000000000E+00
Annihilation of the first spin contaminant:
S**2 before annihilation   2.0282,   after   2.0004

Zero-point correction=   0.243513 (a.u.)
Thermal correction to Energy=   0.264064
Thermal correction to Enthalpy=   0.265008
Thermal correction to Gibbs Free Energy=   0.189859
Sum of electronic and zero-point Energies=   -985.076793
Sum of electronic and thermal Energies=   -985.056242
Sum of electronic and thermal Enthalpies=   -985.055298
Sum of electronic and thermal Free Energies=   -985.130447

	E (Thermal) KCal/Mol	CV Cal/Mol-Kelvin	S Cal/Mol-Kelvin
Total	165.703	72.983	158.165

Item	Value	Threshold	Converged?
Maximum Force	0.000004	0.000450	YES
RMS Force	0.000001	0.000300	YES
Maximum Displacement	0.000319	0.001800	YES
RMS Displacement	0.000053	0.001200	YES

================Figure S5=======================

The base catalyzed reaction of FMF to LF and LF

-------Figure S5-1, FMF + HO(-)(H2O)3, precursor----------

vita-b2.addrev6.high.log

Stoichiometry   C14H19N4O7(1-)

Standard orientation:

Center Number	Atomic Number	Atomic Type	Coordinates (Angstroms) X	Y	Z
1	6	0	-3.591333	-1.954539	-0.096284
2	6	0	-4.441229	-0.958805	0.459909
3	7	0	1.206393	1.559156	-0.769750
4	6	0	1.659053	2.840824	-0.617365
5	7	0	0.776819	3.811950	-0.123364
6	6	0	-0.525204	3.625034	0.256470
7	6	0	-0.989614	2.218212	0.080073
8	7	0	-2.201402	1.923382	0.413896
9	6	0	-2.629953	0.643915	0.244707
10	6	0	-1.791409	-0.348301	-0.299922
11	7	0	-0.494745	0.000409	-0.649249
12	6	0	-0.035345	1.269475	-0.460143
13	6	0	-3.947139	0.312934	0.615326
14	6	0	-2.295477	-1.643788	-0.467039
15	8	0	2.798200	3.172872	-0.888436
16	8	0	-1.217902	4.520818	0.687548
17	6	0	-5.849101	-1.296144	0.863501
18	6	0	0.383978	-1.004150	-1.243229
19	6	0	-4.100598	-3.353774	-0.283621
20	1	0	1.154883	4.745962	-0.029565
21	1	0	-4.560024	1.105125	1.030120
22	1	0	-1.684251	-2.428533	-0.891031
23	1	0	-6.426467	-1.660543	0.009218
24	1	0	-5.862665	-2.084110	1.621662
25	1	0	-6.357241	-0.422000	1.270923
26	6	0	0.834980	-2.054769	-0.243469
27	1	0	-0.118123	-1.480884	-2.085999
28	1	0	1.266847	-0.492352	-1.622123
29	1	0	-4.408458	-3.783445	0.673846
30	1	0	-4.979517	-3.361035	-0.934339
31	1	0	-3.340471	-3.999470	-0.722698
32	8	0	1.351002	-3.079466	-0.613384
33	8	0	2.802728	-0.435257	0.745069
34	1	0	0.642968	-1.841542	0.818913
35	1	0	2.570589	0.304734	0.172403
36	1	0	3.634059	-0.845474	0.364238
37	8	0	4.985976	-1.607925	-0.125547
38	1	0	5.147620	-1.517989	-1.066151
39	1	0	6.136677	-0.952024	0.767117
40	8	0	6.843351	-0.553834	1.364085
41	1	0	6.516394	0.317451	1.591230
42	1	0	4.374165	-3.086603	0.261967
43	8	0	3.956682	-3.955548	0.523761
44	1	0	3.033628	-3.851563	0.271267

Standard basis: 6-311+G(d,p) (6D, 7F)

SCF Done: E(RwB97XD) = -1290.71451358 A.U. after 2 cycles
NFock= 2 Conv=0.18D-08 -V/T= 2.0046
DoSCS=F DFT=T ScalE2(SS,OS)= 1.000000 1.000000

Zero-point correction= 0.337091 (a.u.)
Thermal correction to Energy= 0.365587
Thermal correction to Enthalpy= 0.366531
Thermal correction to Gibbs Free Energy= 0.274076
Sum of electronic and zero-point Energies= -1290.377423
Sum of electronic and thermal Energies= -1290.348926
Sum of electronic and thermal Enthalpies= -1290.347982
Sum of electronic and thermal Free Energies= -1290.440438

	E (Thermal) KCal/Mol	CV Cal/Mol-Kelvin	S Cal/Mol-Kelvin
Total	229.409	101.857	194.589

Item	Value	Threshold	Converged?
Maximum Force	0.000004	0.000450	YES
RMS Force	0.000001	0.000300	YES
Maximum Displacement	0.000366	0.001800	YES
RMS Displacement	0.000085	0.001200	YES

----------Figure S5-2, TS8, C(26)....O(33) formation------

vita-b2.addts6.high.log

Stoichiometry C14H19N4O7(1-)

Standard orientation:

Center Number	Atomic Number	Atomic Type	Coordinates (Angstroms) X	Y	Z
1	6	0	-3.465156	-1.337836	-0.331838
2	6	0	-4.041850	-0.427323	0.598432
3	7	0	1.616572	1.704246	-1.190623
4	6	0	2.278737	2.844285	-0.847406
5	7	0	1.679635	3.726281	0.067486
6	6	0	0.459400	3.582083	0.668234
7	6	0	-0.245341	2.331447	0.265215
8	7	0	-1.408335	2.091834	0.774563
9	6	0	-2.056577	0.957523	0.401475
10	6	0	-1.486010	0.048898	-0.512300
11	7	0	-0.230023	0.325086	-1.024283
12	6	0	0.432864	1.462486	-0.675259
13	6	0	-3.331047	0.694707	0.941953
14	6	0	-2.214911	-1.095816	-0.866963
15	8	0	3.374906	3.137638	-1.293846
16	8	0	0.009613	4.396998	1.445975
17	6	0	-5.399525	-0.693591	1.185835
18	6	0	0.409741	-0.621021	-1.937221
19	6	0	-4.218179	-2.572709	-0.733921
20	1	0	2.210656	4.557128	0.294409
21	1	0	-3.731248	1.418644	1.642739
22	1	0	-1.810929	-1.813107	-1.567428
23	1	0	-6.160685	-0.762982	0.403630
24	1	0	-5.412488	-1.640459	1.733012
25	1	0	-5.688504	0.101606	1.873431
26	6	0	0.875225	-1.911632	-1.270456
27	1	0	-0.298983	-0.878262	-2.727388
28	1	0	1.259591	-0.126204	-2.395953
29	1	0	-4.435098	-3.194521	0.139258
30	1	0	-5.178996	-2.310134	-1.185599
31	1	0	-3.652363	-3.168921	-1.449491
32	8	0	1.494911	-2.722475	-1.950073
33	8	0	2.151172	-0.977042	0.111948
34	1	0	0.349075	-2.210957	-0.355170
35	1	0	2.588570	-0.234997	-0.314405
36	1	0	2.972159	-1.754351	0.623400
37	8	0	3.661464	-2.536385	1.153514
38	1	0	4.571391	-2.341070	0.923599
39	1	0	3.351842	-2.562289	2.826365
40	8	0	3.139835	-2.599813	3.790129
41	1	0	2.884618	-1.706069	4.022852
42	1	0	3.038814	-3.986366	0.332799
43	8	0	2.592219	-4.668084	-0.211056
44	1	0	2.160450	-4.134538	-0.897472

6-311+G(d,p)

SCF Done: E(RwB97XD) = -1290.70687929 A.U. after 1 cycles
NFock= 1 Conv=0.38D-08 -V/T= 2.0046

	1	2	3
	A	A	A
Frequencies --	-751.2610	7.5713	20.0096

Zero-point correction= 0.334434 (a.u.)
Thermal correction to Energy= 0.361952
Thermal correction to Enthalpy= 0.362896
Thermal correction to Gibbs Free Energy= 0.272537
Sum of electronic and zero-point Energies= -1290.372445
Sum of electronic and thermal Energies= -1290.344928
Sum of electronic and thermal Enthalpies= -1290.343984
Sum of electronic and thermal Free Energies= -1290.434342

	E (Thermal) KCal/Mol	CV Cal/Mol-Kelvin	S Cal/Mol-Kelvin
Total	227.128	99.861	190.176

Item	Value	Threshold	Converged?
Maximum Force	0.000003	0.000450	YES
RMS Force	0.000001	0.000300	YES
Maximum Displacement	0.005417	0.001800	NO

RMS Displacement 0.000671 0.001200 YES

-------Figure S5-3, Int5, tetrahedral intermediate-------

vita-b2.addfor6.high.log

Stoichiometry C14H19N4O7(1-)

Standard orientation:

Center Number	Atomic Number	Atomic Type	Coordinates (Angstroms) X	Y	Z
1	6	0	-2.852070	1.502992	-0.847853
2	6	0	-2.572167	2.677474	-0.092485
3	7	0	2.727289	-0.664500	-0.815217
4	6	0	3.992303	-0.409969	-0.387764
5	7	0	4.235783	0.755073	0.362058
6	6	0	3.324909	1.714593	0.704604
7	6	0	1.950058	1.415995	0.210010
8	7	0	1.006532	2.255259	0.483691
9	6	0	-0.246925	1.983578	0.033671
10	6	0	-0.528190	0.816226	-0.703719
11	7	0	0.499265	-0.070289	-0.966485
12	6	0	1.765769	0.187034	-0.537417
13	6	0	-1.283678	2.892793	0.325761
14	6	0	-1.847368	0.599791	-1.137838
15	8	0	4.938276	-1.146258	-0.618905
16	8	0	3.619533	2.701959	1.345741
17	6	0	-3.667824	3.652344	0.235427
18	6	0	0.214876	-1.356961	-1.615035
19	6	0	-4.250125	1.236161	-1.325970
20	1	0	5.191583	0.897347	0.661839
21	1	0	-1.022396	3.773992	0.900773
22	1	0	-2.099210	-0.281748	-1.709738
23	1	0	-4.131472	4.044641	-0.674032
24	1	0	-4.458426	3.172322	0.819107
25	1	0	-3.280881	4.492935	0.811779
26	6	0	-0.444729	-2.374641	-0.652296
27	1	0	-0.428711	-1.184587	-2.476216
28	1	0	1.160168	-1.763869	-1.961132
29	1	0	-4.943705	1.181434	-0.482046
30	1	0	-4.598222	2.045520	-1.973903
31	1	0	-4.308320	0.300264	-1.881200
32	8	0	-0.605839	-3.550269	-1.222134
33	8	0	0.394506	-2.420228	0.564127
34	1	0	-1.373288	-1.926447	-0.239153
35	1	0	1.142989	-2.985375	0.347687
36	1	0	-0.650039	-2.784656	1.877389
37	8	0	-1.452896	-2.889062	2.437210
38	1	0	-1.148890	-3.148897	3.309063
39	1	0	-2.315993	-1.270460	2.258747
40	8	0	-2.756263	-0.411847	2.129872
41	1	0	-2.585685	-0.180020	1.213575
42	1	0	-2.295836	-4.192555	1.144357
43	8	0	-2.472786	-4.663622	0.320499
44	1	0	-1.781030	-4.308472	-0.301085

Standard basis: 6-311+G(d,p) (6D, 7F)

SCF Done: E(RwB97XD) = -1290.72863701 A.U. after 1 cycles
NFock= 1 Conv=0.39D-08 -V/T= 2.0046

Zero-point correction= 0.341436 (a.u.)
Thermal correction to Energy= 0.368702
Thermal correction to Enthalpy= 0.369646
Thermal correction to Gibbs Free Energy= 0.282346
Sum of electronic and zero-point Energies= -1290.387201
Sum of electronic and thermal Energies= -1290.359935
Sum of electronic and thermal Enthalpies= -1290.358991
Sum of electronic and thermal Free Energies= -1290.446291

	E (Thermal) KCal/Mol	CV Cal/Mol-Kelvin	S Cal/Mol-Kelvin
Total	231.364	100.088	183.738

Item	Value	Threshold	Converged?
Maximum Force	0.000032	0.000450	YES
RMS Force	0.000005	0.000300	YES

Maximum Displacement 0.001377 0.001800 YES
RMS Displacement 0.000197 0.001200 YES

-----Figure S5-4, TS9, O(36)H migration and C(18)....N(11) cleavage---

vita-b2.sn2k1.high.log

Stoichiometry C14H19N4O7(1-)

Standard orientation:

Center Number	Atomic Number	Atomic Type	X	Y	Z
1	6	0	3.456768	-1.975828	0.062766
2	6	0	4.411915	-0.912789	-0.019552
3	7	0	-1.410892	1.437906	0.194513
4	6	0	-1.846779	2.701777	0.107732
5	7	0	-0.916655	3.753594	0.030122
6	6	0	0.447068	3.634188	-0.009418
7	6	0	0.906148	2.220009	0.043109
8	7	0	2.182935	1.976149	-0.006124
9	6	0	2.581003	0.683010	0.028940
10	6	0	1.634902	-0.368264	0.123427
11	7	0	0.307091	-0.083944	0.192353
12	6	0	-0.100758	1.189240	0.143815
13	6	0	3.960750	0.379724	-0.036788
14	6	0	2.112215	-1.697457	0.130796
15	8	0	-3.045725	2.998436	0.084900
16	8	0	1.184309	4.598399	-0.085423
17	6	0	5.882050	-1.219936	-0.092667
18	6	0	-1.068443	-1.437237	0.571415
19	6	0	3.929588	-3.401749	0.069619
20	1	0	-1.300456	4.687908	-0.024773
21	1	0	4.652677	1.212204	-0.106078
22	1	0	1.409277	-2.519288	0.180791
23	1	0	6.211853	-1.783984	0.784520
24	1	0	6.115295	-1.827874	-0.971606
25	1	0	6.468608	-0.302448	-0.147961
26	6	0	-1.264217	-1.869108	-0.835506
27	1	0	-0.397075	-1.968158	1.224538
28	1	0	-1.723549	-0.682567	0.973687
29	1	0	4.499399	-3.627256	-0.836328
30	1	0	4.593732	-3.587031	0.918724
31	1	0	3.091649	-4.096279	0.130695
32	8	0	-1.987292	-1.291571	-1.621382
33	1	0	-0.678958	-2.748058	-1.147012
34	8	0	-2.424685	-2.826305	0.780001
35	1	0	-2.006355	-3.538805	1.270339
36	1	0	-3.875957	-1.892032	1.327618
37	8	0	-4.699031	-1.367813	1.376836
38	1	0	-4.581122	-0.686398	0.691450
39	1	0	-3.330495	-0.012230	-1.012147
40	8	0	-4.143595	0.434841	-0.724598
41	1	0	-3.831395	1.294237	-0.392949
42	1	0	-3.740353	-3.436545	-0.266559
43	8	0	-4.607586	-3.624374	-0.681954
44	1	0	-5.178557	-2.997711	-0.226169

Standard basis: 6-311+G(d,p) (6D, 7F)

SCF Done: E(RwB97XD) = -1290.67288847 A.U. after 1 cycles
NFock= 1 Conv=0.74D-08 -V/T= 2.0046

Harmonic frequencies (cm**-1), IR intensities (KM/Mole), Raman

	1	2	3
	A	A	A
Frequencies --	-664.2548	24.4007	29.7397

Zero-point correction= 0.338262 (a.u.)
Thermal correction to Energy= 0.365633
Thermal correction to Enthalpy= 0.366577
Thermal correction to Gibbs Free Energy= 0.280245
Sum of electronic and zero-point Energies= -1290.334626
Sum of electronic and thermal Energies= -1290.307255
Sum of electronic and thermal Enthalpies= -1290.306311
Sum of electronic and thermal Free Energies= -1290.392644

E (Thermal) CV S

```
                 KCal/Mol       Cal/Mol-Kelvin    Cal/Mol-Kelvin
Total              229.438          100.863          181.702

       Item             Value     Threshold  Converged?
Maximum Force           0.000009     0.000450     YES
RMS     Force           0.000001     0.000300     YES
Maximum Displacement     0.000776     0.001800     YES
RMS     Displacement     0.000133     0.001200     YES
```

--------Figure S5-5, [LC-H(+)](-) + HO-CH2-CH=O + (H2)3-------

vita-b2.sn2y.rev.high.log

Stoichiometry   C14H19N4O7(1-)

Standard orientation:

Center Number	Atomic Number	Atomic Type	X	Y	Z
1	6	0	-3.644995	0.425275	-0.924206
2	6	0	-3.993833	-0.896825	-0.484850
3	7	0	2.244357	-0.147658	-0.435890
4	6	0	3.222117	-1.007991	-0.121369
5	7	0	2.893274	-2.316201	0.272988
6	6	0	1.639734	-2.852086	0.385325
7	6	0	0.571357	-1.890125	0.017644
8	7	0	-0.669823	-2.289652	0.096134
9	6	0	-1.632161	-1.404717	-0.238374
10	6	0	-1.285933	-0.100467	-0.667487
11	7	0	0.003631	0.302383	-0.732002
12	6	0	0.952374	-0.563386	-0.392210
13	6	0	-2.997176	-1.775710	-0.158280
14	6	0	-2.327107	0.795238	-1.011246
15	8	0	4.428073	-0.730264	-0.152959
16	8	0	1.453535	-3.999535	0.746886
17	6	0	-5.439520	-1.300284	-0.389412
18	6	0	0.011584	1.224279	2.248932
19	6	0	-4.730171	1.400417	-1.283869
20	1	0	3.675472	-2.916288	0.498569
21	1	0	-3.227919	-2.781891	0.175366
22	1	0	-2.058989	1.792971	-1.342676
23	1	0	-5.986109	-0.650963	0.300670
24	1	0	-5.935921	-1.224799	-1.361315
25	1	0	-5.534402	-2.328019	-0.037492
26	6	0	-1.043256	2.299151	2.093278
27	8	0	-0.521682	3.530445	1.673634
28	1	0	-1.809939	1.908281	1.408198
29	1	0	-5.352953	1.011707	-2.094720
30	1	0	-5.391822	1.579826	-0.431415
31	1	0	-4.311202	2.355864	-1.600256
32	8	0	-0.212657	0.135503	2.718635
33	1	0	1.025900	1.498669	1.893031
34	1	0	-1.525123	2.438596	3.065340
35	1	0	-0.224566	3.443444	0.743895
36	1	0	2.779760	1.542894	-0.469899
37	8	0	3.078812	2.490366	-0.510259
38	1	0	3.392242	2.701370	0.372339
39	1	0	4.697808	1.980557	-1.536523
40	8	0	5.375684	1.337991	-1.787227
41	1	0	5.120365	0.567516	-1.249588
42	1	0	1.358242	3.152703	-0.793285
43	8	0	0.390223	3.091377	-0.869977
44	1	0	0.255094	2.121760	-0.941952

```
Standard basis: 6-311+G(d,p) (6D, 7F)

SCF Done:  E(RwB97XD) =  -1290.75189748  A.U. after 1 cycles
         NFock=  1  Conv=0.40D-08     -V/T= 2.0046

Zero-point correction=                          0.340206 (a.u.)
Thermal correction to Energy=                    0.367906
Thermal correction to Enthalpy=                  0.368850
Thermal correction to Gibbs Free Energy=         0.280090
Sum of electronic and zero-point Energies=          -1290.411692
Sum of electronic and thermal Energies=             -1290.383992
Sum of electronic and thermal Enthalpies=           -1290.383048
Sum of electronic and thermal Free Energies=        -1290.471808
```

	E (Thermal) KCal/Mol	CV Cal/Mol-Kelvin	S Cal/Mol-Kelvin
Total	230.864	100.116	186.812

Item	Value	Threshold	Converged?
Maximum Force	0.000013	0.000450	YES
RMS Force	0.000002	0.000300	YES
Maximum Displacement	0.001028	0.001800	YES
RMS Displacement	0.000228	0.001200	YES

----- Figure S5-6, TS10, C(18)...C(26) cleavage------

vita-b2.w3tsf.high.log

Stoichiometry C14H19N4O7(1-)

Standard orientation:

Center Number	Atomic Number	Atomic Type	Coordinates (Angstroms) X	Y	Z
1	6	0	-3.752850	-1.116515	-0.220242
2	6	0	-4.317280	0.195835	-0.164381
3	7	0	1.967890	0.618622	-0.089297
4	6	0	2.774986	1.669102	0.069671
5	7	0	2.210573	2.954474	0.120165
6	6	0	0.874895	3.260743	0.069294
7	6	0	-0.004267	2.066519	-0.030607
8	7	0	-1.305711	2.245793	-0.034195
9	6	0	-2.064386	1.131401	-0.100042
10	6	0	-1.516450	-0.172153	-0.153695
11	7	0	-0.135934	-0.323845	-0.161949
12	6	0	0.645087	0.810799	-0.091728
13	6	0	-3.475120	1.272020	-0.106201
14	6	0	-2.388457	-1.278066	-0.213697
15	8	0	4.003795	1.568211	0.186018
16	8	0	0.475170	4.410768	0.115330
17	6	0	-5.809508	0.380270	-0.171067
18	6	0	0.499516	-1.550214	-0.049289
19	6	0	-4.653373	-2.318243	-0.282196
20	1	0	2.854045	3.727706	0.225064
21	1	0	-3.865882	2.282839	-0.062041
22	1	0	-1.990463	-2.281664	-0.243156
23	1	0	-6.253838	-0.037037	-1.079404
24	1	0	-6.273859	-0.131185	0.676989
25	1	0	-6.072257	1.437246	-0.117641
26	6	0	0.806685	-1.723878	1.998636
27	1	0	-0.096798	-2.413875	-0.297985
28	1	0	1.521947	-1.526865	-0.393350
29	1	0	-5.306373	-2.362672	0.594436
30	1	0	-5.302471	-2.276828	-1.161707
31	1	0	-4.076520	-3.242418	-0.325582
32	8	0	1.948682	-2.483115	2.026482
33	1	0	1.020099	-0.640161	2.054788
34	8	0	-0.222182	-2.194015	2.506436
35	1	0	2.694969	-1.990947	1.618474
36	8	0	4.171412	-1.345847	0.809294
37	1	0	4.116945	-1.748494	-0.076789
38	1	0	4.073151	-0.394323	0.637743
39	8	0	3.969152	-2.496781	-1.737845
40	1	0	3.201147	-2.210995	-2.266181
41	1	0	3.935360	-3.455567	-1.735899
42	8	0	1.723340	-1.614666	-3.140468
43	1	0	1.013690	-1.558319	-2.491052
44	1	0	1.377569	-2.163905	-3.848490

Standard basis: 6-311+G(d,p) (6D, 7F)

SCF Done: E(RwB97XD) = -1290.69046109 A.U. after 1 cycles
NFock= 1 Conv=0.49D-08 -V/T= 2.0046

Harmonic frequencies (cm**-1), IR intensities (KM/Mole), Raman

	1	2	3
	A	A	A
Frequencies --	-569.8844	16.9488	22.6824

Zero-point correction= 0.337973 (a.u.)
Thermal correction to Energy= 0.365830
Thermal correction to Enthalpy= 0.366775

Thermal correction to Gibbs Free Energy= 0.277400
Sum of electronic and zero-point Energies= -1290.352488
Sum of electronic and thermal Energies= -1290.324631
Sum of electronic and thermal Enthalpies= -1290.323687
Sum of electronic and thermal Free Energies= -1290.413062

	E (Thermal)	CV	S
	KCal/Mol	Cal/Mol-Kelvin	Cal/Mol-Kelvin
Total	229.562	100.890	188.106

Item	Value	Threshold	Converged?
Maximum Force	0.000081	0.000450	YES
RMS Force	0.000014	0.000300	YES
Maximum Displacement	0.003502	0.001800	NO
RMS Displacement	0.000587	0.001200	YES

-------Figure S5-7, [LF-H(+)](-) + HCO2 + (H2O)3--------

vita-b2.w3forf.high.log

Stoichiometry C14H19N4O7(1-)

Standard orientation:

Center Number	Atomic Number	Atomic Type	Coordinates (Angstroms) X	Y	Z
1	6	0	-4.059955	-1.456714	0.149251
2	6	0	-4.693035	-0.175379	0.157445
3	7	0	1.574444	0.607170	-0.089728
4	6	0	2.309462	1.708125	-0.176181
5	7	0	1.668219	2.952544	-0.177384
6	6	0	0.316401	3.173417	-0.111442
7	6	0	-0.488783	1.932942	-0.034058
8	7	0	-1.811724	2.059299	0.023138
9	6	0	-2.492865	0.899372	0.075004
10	6	0	-1.882186	-0.375026	0.085452
11	7	0	-0.480234	-0.470447	0.061860
12	6	0	0.229786	0.735226	-0.025300
13	6	0	-3.912883	0.947356	0.118872
14	6	0	-2.689117	-1.531257	0.115389
15	8	0	3.556535	1.718480	-0.253942
16	8	0	-0.144058	4.304885	-0.121490
17	6	0	-6.193031	-0.073122	0.201000
18	6	0	0.184318	-1.631259	0.085570
19	6	0	-4.890609	-2.710166	0.174553
20	1	0	2.263512	3.767513	-0.239633
21	1	0	-4.364780	1.933786	0.116411
22	1	0	-2.238008	-2.513509	0.101330
23	1	0	-6.647410	-0.572667	-0.659713
24	1	0	-6.594708	-0.553691	1.097970
25	1	0	-6.514286	0.969065	0.199364
26	6	0	3.237919	-1.260935	2.344614
27	1	0	-0.353686	-2.544347	0.262747
28	1	0	1.255915	-1.572230	0.147363
29	1	0	-5.519122	-2.748728	1.069355
30	1	0	-5.561745	-2.756342	-0.688374
31	1	0	-4.260544	-3.600357	0.163377
32	8	0	4.301921	-1.821887	1.810765
33	1	0	2.750900	-0.508159	1.702272
34	8	0	2.821761	-1.554536	3.442017
35	1	0	4.476172	-1.421224	0.900583
36	8	0	4.715901	-0.699604	-0.474877
37	1	0	4.325103	-1.159342	-1.242397
38	1	0	4.277064	0.183451	-0.429846
39	8	0	3.661704	-2.004717	-2.686028
40	1	0	2.690604	-1.966089	-2.787497
41	1	0	3.890619	-2.934182	-2.747444
42	8	0	0.913205	-1.799560	-2.885929
43	1	0	0.575384	-1.781451	-1.972238
44	1	0	0.425815	-2.501413	-3.323973

Standard basis: 6-311+G(d,p) (6D, 7F)

SCF Done: E(RwB97XD) = -1290.71627512 A.U. after 1 cycles
NFock= 1 Conv=0.24D-08 -V/T= 2.0046

Zero-point correction= 0.338077 (a.u.)
Thermal correction to Energy= 0.366545

```
Thermal correction to Enthalpy=              0.367489
Thermal correction to Gibbs Free Energy=       0.275059
Sum of electronic and zero-point Energies=        -1290.378198
Sum of electronic and thermal Energies=           -1290.349731
Sum of electronic and thermal Enthalpies=         -1290.348786
Sum of electronic and thermal Free Energies=       -1290.441216

                E (Thermal)           CV              S
                 KCal/Mol      Cal/Mol-Kelvin   Cal/Mol-Kelvin
Total              230.010          101.760          194.535

      Item             Value    Threshold  Converged?
Maximum Force           0.000014    0.000450    YES
RMS    Force           0.000002    0.000300    YES
Maximum Displacement     0.002994    0.001800    NO
RMS    Displacement    0.000595    0.001200    YES
```

--------Figure S5-8, TS11, formation of the methyl group------

vita-b2.w3tsd.high.log

Stoichiometry C14H19N4O7(1-)

Standard orientation:

Center Number	Atomic Number	Atomic Type	Coordinates (Angstroms) X	Y	Z
1	6	0	-4.013642	-1.395503	0.213428
2	6	0	-4.652008	-0.116824	0.194935
3	7	0	1.597766	0.631745	-0.062248
4	6	0	2.329724	1.742905	-0.211394
5	7	0	1.696947	2.992815	-0.225835
6	6	0	0.349395	3.220965	-0.141876
7	6	0	-0.450852	1.972372	-0.026424
8	7	0	-1.750481	2.081590	0.030408
9	6	0	-2.457179	0.933542	0.112829
10	6	0	-1.832620	-0.332686	0.163710
11	7	0	-0.452629	-0.408693	0.168428
12	6	0	0.268871	0.739031	0.019983
13	6	0	-3.870486	1.005054	0.140607
14	6	0	-2.640842	-1.486275	0.199924
15	8	0	3.561721	1.734272	-0.338813
16	8	0	-0.121773	4.341609	-0.168548
17	6	0	-6.151705	-0.015867	0.221557
18	6	0	0.219003	-1.671491	0.191629
19	6	0	-4.847066	-2.645086	0.241389
20	1	0	2.297835	3.800267	-0.326913
21	1	0	-4.314123	1.994147	0.110246
22	1	0	-2.184055	-2.465023	0.194012
23	1	0	-6.596781	-0.529133	-0.635635
24	1	0	-6.561565	-0.481083	1.122641
25	1	0	-6.472446	1.025996	0.199421
26	6	0	3.568506	-1.254282	2.178758
27	1	0	-0.315039	-2.369275	0.831812
28	1	0	1.236145	-1.520674	0.534658
29	1	0	-5.489507	-2.664409	1.126500
30	1	0	-5.504840	-2.694622	-0.631159
31	1	0	-4.221601	-3.537749	0.251623
32	8	0	4.470714	-1.896830	1.468657
33	1	0	3.035784	-0.463656	1.625015
34	8	0	3.334102	-1.514935	3.336223
35	1	0	4.510455	-1.506100	0.539248
36	8	0	4.550879	-0.774121	-0.856416
37	1	0	3.976432	-1.181892	-1.539019
38	1	0	4.192346	0.126192	-0.705768
39	8	0	3.014861	-1.934631	-2.798322
40	1	0	2.052863	-2.136219	-2.625820
41	1	0	3.040053	-1.476529	-3.640131
42	8	0	0.470203	-2.419311	-2.298756
43	1	0	0.321816	-2.131065	-1.167550
44	1	0	0.194768	-3.334926	-2.384975

Standard basis: 6-311+G(d,p) (6D, 7F)

```
SCF Done:  E(RwB97XD) =  -1290.70948210 A.U. after 1 cycles
         NFock=  1  Conv=0.51D-08     -V/T= 2.0046
```

Harmonic frequencies (cm**-1), IR intensities (KM/Mole), Raman

	1	2	3
	A	A	A
Frequencies --	-1323.9417	16.6905	21.1656

Zero-point correction= 0.333828 (a.u.)
Thermal correction to Energy= 0.361042
Thermal correction to Enthalpy= 0.361986
Thermal correction to Gibbs Free Energy= 0.273344
Sum of electronic and zero-point Energies= -1290.375654
Sum of electronic and thermal Energies= -1290.348440
Sum of electronic and thermal Enthalpies= -1290.347496
Sum of electronic and thermal Free Energies= -1290.436138

	E (Thermal)	CV	S
	KCal/Mol	Cal/Mol-Kelvin	Cal/Mol-Kelvin
Total	226.557	98.364	186.564

Item	Value	Threshold	Converged?
Maximum Force	0.000007	0.000450	YES
RMS Force	0.000001	0.000300	YES
Maximum Displacement	0.001137	0.001800	YES
RMS Displacement	0.000132	0.001200	YES

----Figure S5-9, LF + HCO2(-) + (H2O)3------

vita-b2.w3revd.high.log

Stoichiometry C14H19N4O7(1-)

Standard orientation:

Center Number	Atomic Number	Atomic Type	X	Y	Z
1	6	0	3.918950	-1.457216	0.217512
2	6	0	4.528137	-0.203513	-0.068956
3	7	0	-1.731243	0.382526	-0.363813
4	6	0	-2.500717	1.473900	-0.580475
5	7	0	-1.893170	2.700750	-0.866436
6	6	0	-0.548661	2.960480	-0.857768
7	6	0	0.281400	1.752246	-0.585693
8	7	0	1.566064	1.872782	-0.585616
9	6	0	2.315938	0.767392	-0.319080
10	6	0	1.719895	-0.475303	-0.029737
11	7	0	0.339821	-0.564073	-0.020953
12	6	0	-0.426600	0.513720	-0.326026
13	6	0	3.719722	0.875525	-0.330756
14	6	0	2.542475	-1.578583	0.234613
15	8	0	-3.727752	1.434748	-0.539322
16	8	0	-0.096989	4.068158	-1.058611
17	6	0	6.025507	-0.069071	-0.085505
18	6	0	-0.305365	-1.834488	0.322195
19	6	0	4.774900	-2.657923	0.501682
20	1	0	-2.514788	3.484722	-1.016260
21	1	0	4.141801	1.848678	-0.555454
22	1	0	2.106589	-2.544764	0.447750
23	1	0	6.456118	-0.338776	0.882984
24	1	0	6.472662	-0.731504	-0.832154
25	1	0	6.321873	0.954270	-0.317155
26	6	0	-3.244261	-2.885285	-1.274256
27	1	0	-0.111381	-2.568655	-0.460825
28	1	0	-1.372268	-1.661243	0.407945
29	1	0	5.435857	-2.872015	-0.342962
30	1	0	5.414757	-2.480523	1.370832
31	1	0	4.167591	-3.541795	0.696343
32	8	0	-3.707041	-3.067732	-0.116142
33	1	0	-3.069917	-1.821830	-1.557918
34	8	0	-2.956793	-3.753358	-2.117544
35	1	0	-4.038330	-1.627412	0.725289
36	8	0	-4.241417	-0.788053	1.207770
37	1	0	-2.909205	-0.428495	2.261752
38	1	0	-4.142101	-0.087930	0.542025
39	8	0	-2.172476	-0.188674	2.871029
40	1	0	-1.333032	1.337635	2.464185
41	1	0	-2.541779	-0.240928	3.754612
42	8	0	-0.833231	2.146464	2.237977
43	1	0	0.088348	-2.188775	1.273814
44	1	0	-1.465423	2.861719	2.325166

Standard basis: 6-311+G(d,p) (6D, 7F)

SCF Done: E(RwB97XD) = -1290.76254086 A.U. after 2 cycles
NFock= 2 Conv=0.84D-09 -V/T= 2.0046

Zero-point correction=	0.339188 (a.u.)
Thermal correction to Energy=	0.368127
Thermal correction to Enthalpy=	0.369071
Thermal correction to Gibbs Free Energy=	0.276799
Sum of electronic and zero-point Energies=	-1290.423352
Sum of electronic and thermal Energies=	-1290.394414
Sum of electronic and thermal Enthalpies=	-1290.393470
Sum of electronic and thermal Free Energies=	-1290.485741

	E (Thermal) KCal/Mol	CV Cal/Mol-Kelvin	S Cal/Mol-Kelvin
Total	231.003	101.516	194.202

Item	Value	Threshold	Converged?
Maximum Force	0.000017	0.000450	YES
RMS Force	0.000003	0.000300	YES
Maximum Displacement	0.002060	0.001800	NO
RMS Displacement	0.000481	0.001200	YES

==============Figure S6 ==================

A hydrogen-shift reaction between the
triplet lumiflavin,LF(T),and
(R)-2-amino-(S)-4-hydroxy-(R)-5-(hydroxymethyl)-tetrahydrofuran,
"subst.THF".

---------FigureS6, before reaction, LF(T).....subst.THF------

lf-hfor2c.high.log

Stoichiometry C18H23N5O5(3)

Standard orientation:

Center Number	Atomic Number	Atomic Type	Coordinates (Angstroms) X	Y	Z
1	6	0	-0.289963	3.599998	-0.096549
2	6	0	0.695522	2.718427	0.452464
3	7	0	-4.613564	-0.490976	-0.396592
4	6	0	-4.943203	-1.821997	-0.190161
5	7	0	-3.964629	-2.657344	0.304755
6	6	0	-2.666787	-2.293605	0.627861
7	6	0	-2.362228	-0.883879	0.408018
8	7	0	-1.123810	-0.447797	0.704520
9	6	0	-0.889976	0.867779	0.447536
10	6	0	-1.883998	1.763791	-0.097749
11	7	0	-3.135865	1.282695	-0.352756
12	6	0	-3.405421	-0.078557	-0.112738
13	6	0	0.372632	1.395366	0.712992
14	6	0	-1.547264	3.109815	-0.354181
15	8	0	-6.059958	-2.240987	-0.439148
16	8	0	-1.874006	-3.116892	1.056757
17	6	0	2.075609	3.216280	0.739697
18	6	0	-4.178109	2.159993	-0.881401
19	6	0	0.050075	5.032486	-0.384769
20	1	0	-4.217326	-3.626667	0.445202
21	1	0	1.126361	0.727017	1.111622
22	1	0	-2.291857	3.775957	-0.767835
23	1	0	2.552752	3.576810	-0.177132
24	1	0	2.046628	4.062401	1.432840
25	1	0	2.695153	2.430260	1.169485
26	1	0	-4.340861	2.990076	-0.193056
27	1	0	-5.091224	1.585289	-0.980867
28	1	0	-3.878919	2.540902	-1.858970
29	1	0	0.359142	5.553162	0.526228
30	1	0	0.881039	5.103113	-1.092580
31	1	0	-0.804507	5.560880	-0.807095
32	1	0	1.377710	-0.859503	-0.827597
33	6	0	2.041949	-1.547491	-0.292987
34	8	0	2.834894	-0.688563	0.563302
35	6	0	3.037638	-2.223080	-1.242066
36	6	0	4.207468	-0.948481	0.356855
37	6	0	4.294503	-1.379046	-1.108966

38	1	0	2.656689	-2.283904	-2.261385
39	7	0	1.296940	-2.490139	0.497907
40	1	0	0.377053	-2.120907	0.730300
41	1	0	1.173438	-3.367322	0.008825
42	6	0	5.009254	0.296220	0.676367
43	8	0	6.361335	-0.005318	0.343453
44	1	0	6.892465	0.787375	0.432992
45	1	0	4.633816	1.129274	0.070422
46	1	0	4.914027	0.557689	1.735679
47	1	0	4.553488	-1.782582	0.986810
48	8	0	5.459182	-2.114050	-1.422181
49	1	0	6.203260	-1.572665	-1.134472
50	1	0	4.226952	-0.485122	-1.745021
51	1	0	3.263435	-3.234234	-0.888032

```
---------------------------------------------------------------------
  782 basis functions
  104 alpha electrons     102 beta electrons
     nuclear repulsion energy     2674.9965144184 Hartrees.
NAtoms=   51 NActive=   51

SCF Done:  E(UwB97XD) =  -1349.59356983  A.U. after 1 cycles
         NFock=  1  Conv=0.38D-08    -V/T= 2.0048

Zero-point correction=                       0.412286 (a.u.)
Thermal correction to Energy=                 0.440248
Thermal correction to Enthalpy=               0.441192
Thermal correction to Gibbs Free Energy=       0.348474
Sum of electronic and zero-point Energies=        -1349.181283
Sum of electronic and thermal Energies=           -1349.153322
Sum of electronic and thermal Enthalpies=         -1349.152378
Sum of electronic and thermal Free Energies=       -1349.245096

                 E (Thermal)           CV             S
                  KCal/Mol       Cal/Mol-Kelvin   Cal/Mol-Kelvin
Total               276.260          103.705          195.142

       Item             Value    Threshold  Converged?
Maximum Force           0.000004    0.000450    YES
RMS    Force           0.000001    0.000300    YES
Maximum Displacement    0.006026    0.001800    NO
RMS    Displacement    0.001267    0.001200    NO

   -------Figure S6, TS of the H(32) migration-------

 lf-hts2c.high.log

 Stoichiometry   C18H23N5O5(3)

                     Standard orientation:
```

Center Number	Atomic Number	Atomic Type	Coordinates (Angstroms) X	Y	Z
1	6	0	-3.624123	1.840578	0.082996
2	6	0	-2.484441	2.483639	0.609270
3	7	0	-1.156475	-3.624112	-0.435312
4	6	0	-0.021473	-4.328424	-0.233291
5	7	0	1.095103	-3.654067	0.276374
6	6	0	1.135725	-2.322941	0.587495
7	6	0	-0.075266	-1.602344	0.371594
8	7	0	-0.078637	-0.287573	0.679605
9	6	0	-1.248429	0.378193	0.486388
10	6	0	-2.398986	-0.259800	-0.038092
11	7	0	-2.336954	-1.624865	-0.354021
12	6	0	-1.176355	-2.325593	-0.143703
13	6	0	-1.331506	1.746605	0.802597
14	6	0	-3.562581	0.484937	-0.228495
15	8	0	0.090698	-5.532627	-0.471676
16	8	0	2.180936	-1.801202	1.033438
17	6	0	-2.518123	3.949492	0.950697
18	6	0	-3.515286	-2.285107	-0.896612
19	6	0	-4.899317	2.606595	-0.143277
20	1	0	1.933893	-4.199614	0.417041
21	1	0	-0.435380	2.214176	1.194757
22	1	0	-4.448785	0.009616	-0.628700
23	1	0	-2.763875	4.555426	0.073179
24	1	0	-3.277287	4.164293	1.709081
25	1	0	-1.552592	4.283392	1.333516
26	1	0	-4.342227	-2.226513	-0.185134
27	1	0	-3.268425	-3.325253	-1.078410

28	1	0	-3.812914	-1.811589	-1.834910
29	1	0	-5.270070	3.043535	0.789214
30	1	0	-4.745280	3.434511	-0.842472
31	1	0	-5.680616	1.962379	-0.549084
32	1	0	1.910047	-0.088753	1.151647
33	6	0	2.618591	0.906113	1.335761
34	8	0	1.988198	1.999965	0.810136
35	6	0	3.841392	0.592061	0.472094
36	6	0	2.403446	2.186241	-0.551540
37	6	0	3.287193	0.974240	-0.890401
38	1	0	4.151061	-0.445517	0.555283
39	7	0	2.733875	0.955627	2.707623
40	1	0	2.018745	1.458318	3.217426
41	1	0	3.080827	0.121962	3.161963
42	6	0	1.198904	2.326335	-1.459444
43	8	0	1.743441	2.468045	-2.765639
44	1	0	1.037121	2.397356	-3.409628
45	1	0	0.578613	1.427716	-1.380386
46	1	0	0.597760	3.197496	-1.179445
47	1	0	3.016453	3.093167	-0.591324
48	8	0	4.310249	1.274830	-1.806287
49	1	0	3.878705	1.576964	-2.612741
50	1	0	2.654737	0.161321	-1.271554
51	1	0	4.656727	1.263513	0.756796

SCF Done: E(UwB97XD) = -1349.57929309 A.U. after 1 cycles

Harmonic frequencies (cm**-1), IR intensities (KM/Mole), Raman

	1	2	3
	A	A	A
Frequencies --	-250.0438	13.8640	16.1587

Zero-point correction=	0.408939 (a.u.)
Thermal correction to Energy=	0.435620
Thermal correction to Enthalpy=	0.436564
Thermal correction to Gibbs Free Energy=	0.350301
Sum of electronic and zero-point Energies=	-1349.170354
Sum of electronic and thermal Energies=	-1349.143673
Sum of electronic and thermal Enthalpies=	-1349.142729
Sum of electronic and thermal Free Energies=	-1349.228992

	E (Thermal)	CV	S
	KCal/Mol	Cal/Mol-Kelvin	Cal/Mol-Kelvin
Total	273.355	102.212	181.555

Item	Value	Threshold	Converged?
Maximum Force	0.000010	0.000450	YES
RMS Force	0.000001	0.000300	YES
Maximum Displacement	0.000693	0.001800	YES
RMS Displacement	0.000128	0.001200	YES

------Figure S6, after reaction, [LF+H](.)....[subst.THF-H](.)----

lf-hrev2c.high.log

Stoichiometry C18H23N5O5(3)

Standard orientation:

Center Number	Atomic Number	Atomic Type	Coordinates (Angstroms) X	Y	Z
1	6	0	0.502240	3.733897	0.158091
2	6	0	1.410359	2.784259	0.676098
3	7	0	-4.142320	-0.026192	-0.546221
4	6	0	-4.550854	-1.310353	-0.388486
5	7	0	-3.632991	-2.245411	0.107702
6	6	0	-2.332229	-1.990674	0.461659
7	6	0	-1.948872	-0.616916	0.265375
8	7	0	-0.686237	-0.228301	0.569151
9	6	0	-0.291044	1.078670	0.444265
10	6	0	-1.206779	2.016491	-0.056377
11	7	0	-2.499577	1.606842	-0.404787
12	6	0	-2.900418	0.304427	-0.232540
13	6	0	0.997802	1.472914	0.812944
14	6	0	-0.782750	3.340390	-0.191990
15	8	0	-5.684004	-1.697545	-0.655982
16	8	0	-1.585897	-2.864278	0.902016
17	6	0	2.803866	3.186799	1.072732

18	6	0	-3.430156	2.593705	-0.942369
19	6	0	0.918028	5.169147	-0.008920
20	1	0	-3.967188	-3.192717	0.220975
21	1	0	1.669462	0.721550	1.215550
22	1	0	-1.463743	4.086311	-0.578465
23	1	0	3.366103	3.558067	0.210492
24	1	0	2.786869	3.989765	1.814894
25	1	0	3.350408	2.344022	1.497514
26	1	0	-3.605262	3.381184	-0.206932
27	1	0	-4.364744	2.092078	-1.165590
28	1	0	-3.020613	3.029030	-1.855469
29	1	0	1.202814	5.609596	0.951230
30	1	0	1.786860	5.251530	-0.668539
31	1	0	0.110919	5.767976	-0.431800
32	1	0	0.019900	-0.938190	0.832316
33	6	0	1.612853	-2.168160	1.003177
34	8	0	2.803497	-1.471795	1.146308
35	6	0	1.573057	-2.843923	-0.338349
36	6	0	3.522947	-1.519988	-0.088515
37	6	0	2.492499	-1.939830	-1.145936
38	1	0	0.563543	-2.923684	-0.742439
39	7	0	1.210767	-2.842448	2.158753
40	1	0	1.339523	-2.285225	2.995386
41	1	0	0.237332	-3.122747	2.077603
42	6	0	4.176399	-0.182955	-0.369477
43	8	0	4.827236	-0.336815	-1.625007
44	1	0	5.152524	0.515951	-1.917791
45	1	0	3.408188	0.596670	-0.422860
46	1	0	4.890328	0.074768	0.419811
47	1	0	4.290091	-2.301682	-0.021339
48	8	0	3.067143	-2.609308	-2.244723
49	1	0	3.723916	-2.010322	-2.616758
50	1	0	1.943094	-1.051828	-1.488312
51	1	0	2.006698	-3.852993	-0.280856

SCF Done: E(UwB97XD) = -1349.62582565 A.U. after 1 cycles

Zero-point correction= 0.413776 (a.u.)
Thermal correction to Energy= 0.441231
Thermal correction to Enthalpy= 0.442175
Thermal correction to Gibbs Free Energy= 0.354204
Sum of electronic and zero-point Energies= -1349.212049
Sum of electronic and thermal Energies= -1349.184595
Sum of electronic and thermal Enthalpies= -1349.183650
Sum of electronic and thermal Free Energies= -1349.271621

	E (Thermal) KCal/Mol	CV Cal/Mol-Kelvin	S Cal/Mol-Kelvin
Total	276.877	103.523	185.150

Item	Value	Threshold	Converged?
Maximum Force	0.000003	0.000450	YES
RMS Force	0.000000	0.000300	YES
Maximum Displacement	0.000610	0.001800	YES
RMS Displacement	0.000118	0.001200	YES
